# DNA methylation signature on phosphatidylethanol, not on self-reported alcohol consumption, predicts hazardous alcohol consumption in two distinct populations

**DOI:** 10.1038/s41380-020-0668-x

**Published:** 2020-02-07

**Authors:** Xiaoyu Liang, Amy C. Justice, Kaku So-Armah, John H. Krystal, Rajita Sinha, Ke Xu

**Affiliations:** 1 Department of Psychiatry, Yale School of Medicine, New Haven, CT USA; 2 VA Connecticut Healthcare System, West Haven, CT USA; 3 Yale School of Medicine, New Haven, CT USA; 4 Boston University School of Medicine, Boston, MA USA; 5 Child Study Center, Yale School of Medicine, New Haven, CT USA; 6 Stress Center, Yale School of Medicine, New Haven, CT USA

**Keywords:** Genetics, Biomarkers

## Abstract

The process of diagnosing hazardous alcohol drinking (HAD) is based on self-reported data and is thereby vulnerable to bias. There has been an interest in developing epigenetic biomarkers for HAD that might complement clinical assessment. Because alcohol consumption has been previously linked to DNA methylation (DNAm), we aimed to select DNAm signatures in blood to predict HAD from two demographically and clinically distinct populations (*N*_total_ = 1,549). We first separately conducted an epigenome-wide association study (EWAS) for phosphatidylethanol (PEth), an objective measure of alcohol consumption, and for self-reported alcohol consumption in Cohort 1. We identified 83 PEth-associated CpGs, including 23 CpGs previously associated with alcohol consumption or alcohol use disorder. In contrast, no CpG reached epigenome-wide significance on self-reported alcohol consumption. Using a machine learning approach, two CpG subsets from EWAS on PEth and on self-reported alcohol consumption from Cohort 1 were separately tested for the prediction of HAD in Cohort 2. We found that a subset of 143 CpGs selected from the EWAS on PEth showed an excellent prediction of HAD with the area under the receiver operating characteristic curve (AUC) of 89.4% in training set and 73.9% in validation set of Cohort 2. However, CpGs preselected from the EWAS on self-reported alcohol consumption showed a poor prediction of HAD with AUC 75.2% in training set and 57.6% in validation set. Our results demonstrate that an objective measure for alcohol consumption is a more informative phenotype than self-reported data for revealing epigenetic mechanisms. The PEth-associated DNAm signature in blood could serve as a robust biomarker for alcohol consumption.

## Introduction

Hazardous alcohol drinking (HAD) is detrimental to health and is highly correlated with medical comorbidities and psychiatric diseases [1, 2]. Diagnosing HAD is challenging due to a lack of stable and objective measures for chronic heavy alcohol consumption [3]. Phosphatidylethanol (PEth) is a lipid metabolite of ethanol formed from phosphatidylcholine in erythrocytes and has been proposed as a biomarker for alcohol consumption. Compared with self-reported data, PEth reliably detects ethanol levels up to 21 days after the last drink [4], and the PEth level is highly correlated with alcohol consumption [5]. However, the clinical applicability of PEth is limited because its half-life is ~4–7 days and its window of detection is considered to be 21 days [6]. Thus, other longer-term biomarkers for alcohol consumption are needed to inform clinical practice.

Epigenetic signatures have emerged as attractive biomarkers for complex diseases such as cancers and neurodegenerative diseases [7]. Epigenetic markers may reflect environmental exposures, including alcohol consumption. Among these epigenetic markers, DNA methylation (DNAm) biomarkers are particularly attractive because they are relatively stable and capture an early stage of pathophysiological changes [8, 9]. A recent longitudinal study on DNAm showed that most DNA methylome changes occurred 80–90 days before clinically detectable glucose elevation [10], suggesting that DNAm is involved in an early stage of diabetes. Finally, epigenetic modifications can be reliably detected in noninvasive fluids and biospecimens [11]. Thus, the utility of epigenetic alterations has motivated the biomarker research field to develop epigenetic signatures derived from easily accessible cells for clinical use [12–14].

DNAm markers are emerging as diagnostic biomarkers in many areas of medicine and are applied to predict complex diseases [15]. For example, DNAm markers on the promoters of several genes, including *BMP3, NDRG4*, and *SPEPT9*, in blood or stool samples have been approved by the Food and Drug Administration as biomarkers for colorectal cancer screening [16]. DNAm markers also distinguish smokers and nonsmokers [17, 18]. However, we do not yet have validated DNAm biomarkers for the diagnosis of HAD.

DNAm is directly altered by HAD in the following manner. HAD often causes folate and vitamin B deficiency, resulting in the reduction of S-adenosylmethionine (SAM). DNAm is modulated by DNA methyltransferase which transfers a methyl group from SAM to the 5-position of cytosine in the context of cytosine-phosphate-guanine (CpG) dinucleotide. Reduced methyl transfer reaction co-factors (folate and vitamin B) reduce methyltransferase activity that may lead to alteration in DNAm. Recent studies have shown that alcohol consumption modifies DNAm [19] in animals and in the human epigenome from blood, liver, and saliva cells [17, 20–24]. As a result, DNAm in peripheral cells can serve as a robust biomarker for HAD.

Epigenome-wide association studies (EWAS) have identified hundreds of DNAm CpGs in blood for alcohol consumption [25–28], alcohol use disorder (AUD) [29, 30], stress-related alcohol consumption [31], and fetal alcohol syndrome [32–35]. A large number of CpGs in the blood have recently been reported to have associations with dietary folate and alcohol intake [36]. CpGs have been associated with alcohol consumption in different cell types, ethnic groups, and phenotypic assessments [28, 29, 37]. More than a dozen CpGs for alcohol phenotypes have been replicated. For example, cg11376147 on *SLC43A1* has been linked to alcohol consumption and HAD diagnosis in several studies [17, 28, 29]. Thus, DNAm in blood has been proposed as a diagnostic and prognostic biomarker of alcohol consumption for clinical use [38]. For this purpose, a previous study identified a panel of 144 CpGs as biomarkers for alcohol consumption [29]. However, these CpGs have not been validated in independent studies.

Differentially methylated CpG sites have also been associated with differential gene expression for alcohol exposure in both animals and humans. Alcohol exposure is associated with hypomethylation in the promoter of the proprotein convertase subtilisin/kexin type 9 (*PCSK9*) gene [30] that is also correlated with *PCSK9* expression for heavy alcohol consumption in humans and mice. Because *PCSK9* is well known to regulate low-density lipoprotein cholesterol, DNAm alteration and dysfunction of *PCSK9* is thought to be a mechanism for alcohol-related abnormalities in lipid metabolism. Most recently, Gatta et al. [31] reported the hypermethylation of DNA 5-methylcytosine at the promoter regions of *NR3C1* (Nuclear Receptor Subfamily 3 Group C Member 1), the glucocorticoid receptor, that was correlated with the reduction of mRNA expression of *NR3C1* in human brains with AUD. The expression levels of several stress-responsive genes within the *NR3C1* gene network were also decreased in brain samples from individuals with AUD. This evidence further supports the feasibility of DNAm biomarkers for HAD that may have both clinical utilities and help elucidate underlying pathophysiologic mechanisms of heavy alcohol consumption.

One of the limitations of previous EWAS is that alcohol consumption was assessed by self-report, which may lead to inaccurate assessment and introduce bias [29, 39, 40]. A self-reported phenotype may, in part, explain the discrepancy of EWAS findings on alcohol consumption or alcohol use-related phenotypes observed in previous studies. Objective measures such as PEth may improve the association signals for alcohol consumption in EWAS because PEth-associated DNAm markers are more proximal to the biological changes and pathological processes underlying HAD.

**Fig. 1:**
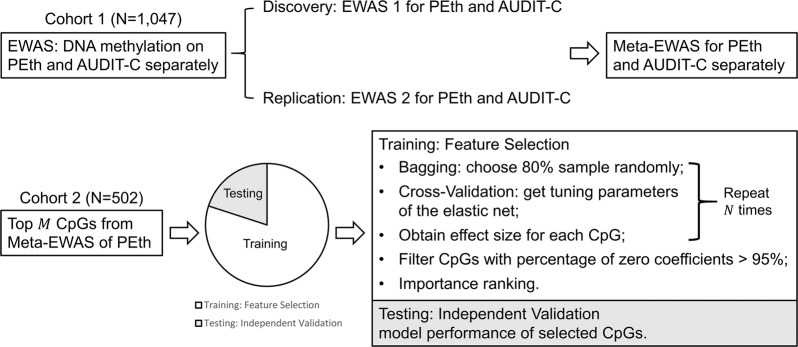
Study design for the epigenome-wide association study for alcohol consumption.

In this study, we hypothesized that the DNAm signatures associated with PEth would be a more robust predictor of HAD than self-reported drinking data. We conducted a 2-stage study with the goal of identifying DNAm CpGs for alcohol consumption and linking the CpG features to HAD (*N*_total_ = 1,549). We first identified CpGs associated with PEth in Cohort 1. Then, the informative CpGs were selected to predict HAD by using elastic net regularization (ENR) in a demographically and clinically independent sample (Cohort 2). We also compared the findings of DNAm markers for PEth with those for self-reported alcohol consumption. The analytical strategy is presented in Fig. 1.

## Materials and methods

### Sample descriptions

Cohort 1 (*N* = 1,047): The DNA samples in Cohort 1 were from the Veterans Aging Cohort Study (VACS) [41]. Data were obtained from the patients after they provided written consent; data were collected via telephone interviews, focus groups, and full access to the national Veterans Affairs electronic medical record system. All subjects in this subset of the VACS cohort were men.

Samples in Cohort 1 were divided into a discovery set (*N* = 580) and a replication set (*N* = 467) for EWAS. A majority of discovery samples were HIV-positive (~85.34%), and all replication samples were HIV-positive.

Cohort 2 (*N* = 502): We recruited 502 HIV-negative healthy community volunteers who responded to advertisements placed either online or in local newspapers and at a community center in New Haven, CT [42]. Phenotypic assessment including alcohol consumption was obtained through the in-person interview. To reduce confounding effects, we excluded subjects who met the Diagnostic and Statistical Manual of Mental Disorders, 4th Edition (DSM-IVTR) (American Psychiatric Association, 1994) criteria for substance dependence on any drug or alcohol other than nicotine. Subjects with head injury or those who used prescribed medications for any psychiatric or medical disorders were also excluded.

All phenotypic data in Cohort 1 and Cohort 2 were obtained in the same time window as the blood draws for DNAm profiling. Genomic DNA was extracted from whole blood using a standard method [43]. The study was approved by the committee of the Human Research Subject Protection at Yale University and the IRB committee of the Connecticut Veteran Healthcare System.

### Phosphatidylethanol (PEth) measurement

In this study, PEth was only measured in Cohort 1 using dried blood spot samples derived from frozen peripheral blood mononuclear cells stored at −80 °C [5]. We analyzed the PEth levels from dry blood spots at the U.S. Drug Testing Laboratory (Des Plaines, IL) via LC-MS/MS, as described in previous studies [44–46]. The LC-MS/MS method has a high capacity and is cost-effective and clinically reliable [46]. PEth can be detected at concentrations as low as 2 ng/ml. A study showed that the PEth value is linearly related to alcohol consumption [47]. In forensics, 20 ng/ml of PEth was used as a cutoff to detect harmful alcohol use [48]. The sensitivity of PEth has been reported to be 99% [47], with several studies showing the assay to have perfect specificity, including in the presence of liver disease and hypertension. We previously reported that PEth was highly correlated with the Alcohol Use Disorders Identification Test-Consumption (AUDIT-C, first three items of AUDIT) score from electronic records [49].

### Definition of hazardous alcohol drinking (HAD)

Alcohol consumption was measured by both PEth and AUDIT-C in Cohort 1 and was only measured by AUDIT in Cohort 2 (Supplementary Table S1). In Cohort 1, HAD was defined as PEth level ≥20 based on previous studies and non-HAD was defined as PEth <20 [48]. HAD was corresponding to AUDIT-C score ≥4 and non-HAD was corresponding to AUDIT-C score <4 for men [5]. In the discovery set of Cohort 1, there were 166 HADs and 414 non-HADs. In the replication set of Cohort 1, there were 135 HADs and 332 non-HADs. In Cohort 2, alcohol consumption was assessed by a full scale of 10-item AUDIT with a total score of 40. HAD was defined as AUDIT ≥8 for men and ≥7 for women based on previous studies. Non-HAD was defined as AUDIT <8 for men and <7 for women [50]. There were 148 HADs and 354 non-HADs. Cohort 2 was divided into a training set (*N* = 402) and a testing set (*N* = 100), with an 80–20 split, for machine learning prediction of HAD. Demographic and clinical variables for HAD versus non-HAD participants in Cohort 1 and Cohort 2 are presented in Table 1.

**Table 1:** Demographic and clinical characterizations for Cohort 1 and Cohort 2.

	Cohort 1: discovery sample		Cohort 1: replication sample		Cohort 2	
	HAD PEth ≥ 20 (*N* = 166)	non-HAD PEth < 20 (*N* = 414)	HAD PEth ≥ 20 (*N* = 135)	non-HAD PEth < 20 (*N* = 332)	HAD Men: AUDIT ≥ 8 Women: AUDIT ≥ 7 (*N* = 148)	non-HAD Men: AUDIT < 8 Women: AUDIT < 7 (*N* = 354)
Age (year)	49.28 ± 7.25	49.25 ± 8.13	47.50 ± 7.08	48.18 ± 8.03	26.80 ± 7.13	29.76 ± 9.28^a^
Sex (male, %)	100	100	100	100	64.86	35.31^b^
Race (AA, %)	90.36	79.71^c1^	82.22	81.02	12.24	22.38^c2^
Smoker (%)	70.91	53.92^d1^	63.64	54.91	39.86	12.71^d2^
Alcohol (AUDIT-C)	4.73 ± 2.65	2.57 ± 2.40^e1^	4.80 ± 2.30	2.28 ± 2.24^e2^	NA	NA
HIV-infection (%)	88.55	84.54	100	100	NA	NA
VL (log10)	2.85 ± 1.24	2.6 ± 1.2	2.69 ± 1.20	2.68 ± 1.24	NA	NA
ART adherence (%)	69.23	81.69^f^	72.73	77.2	NA	NA
CD4+ T (%)	0.06 ± 0.06	0.07 ± 0.06	0.10 ± 0.05	0.09 ± 0.04	0.18 ± 0.05	0.18 ± 0.05
CD8+ T (%)	0.17 ± 0.09	0.16 ± 0.09	0.18 ± 0.09	0.18 ± 0.08	0.10 ± 0.04	0.09 ± 0.04
NK (%)^g^	0.07 ± 0.05	0.08 ± 0.06	0.09 ± 0.03	0.08 ± 0.03	0.03 ± 0.03	0.03 ± 0.03
B cell (%)^g^	0.08 ± 0.05	0.09 ± 0.05^h^	0.08 ± 0.03	0.08 ± 0.04	0.07 ± 0.03	0.07 ± 0.03
Monocyte (%)^g^	0.12 ± 0.04	0.11 ± 0.04	0.11 ± 0.04	0.11 ± 0.03	0.08 ± 0.02	0.08 ± 0.02
Granulocyte (%)^g^	0.53 ± 0.12	0.53 ± 0.14	0.50 ± 0.11	0.50 ± 0.12	0.58 ± 0.09	0.59 ± 0.09

*AA* African American, *AUDIT* Alcohol Use Disorders Identification Test, *AUDIT-C* first three questions of the Alcohol Use Disorders Identification Test, *VL* viral load, *ART* antiretroviral therapy.

^a^Welch’s two-sample *t*-test (degrees of freedom (df) = 360) *P* value = 1.35E−04.

^b^Chi-square test *P* value = 2.18E−09.

^c1^Chi-square test *P* value = 3.20E−03.

^c2^Chi-square test *P* value = 1.29E−02.

^d1^Chi-square test *P* value = 2.65E−04.

^d2^Chi-square test *P* value = 1.77E−11.

^e1^Welch’s two-sample *t*-test (df=280) *P* value = 3.50E−14.

^e2^Welch’s two-sample *t*-test (df=240) *P* value = 4.27E−18.

^f^Chi-square test *P* value = 3.69E−03.

^g^Cell-type compositions estimated by methylation.

^h^Welch’s two-sample *t*-test (df=320) *P* value = 2.34E−02.

### DNA methylation and data quality control (QC)

In Cohort 1, DNAm for the discovery sample was profiled by using the Illumina Infinium HumanMethylation450 Beadchip (Illumina HM450K) (San Diego, CA, USA). DNAm for the replication sample was assessed by using the Illumina Infinium MethylationEPIC Beadchip (Illumina EPIC) (San Diego, CA, USA). In Cohort 2, DNAm was measured by using Illumina HM450K. All samples in Cohorts 1 and 2 were processed at the Yale Center for Genomic Analysis [14]. After QC (details in Supplementary Information), in Cohort 1, a total of 437,722 CpGs from 450K array remained in the discovery sample and 846,604 CpGs from EPIC array remained for the replication sample. A total of 48.26% common CpGs (408,583) were analyzed in meta-analyses. In Cohort 2, we applied the same QC criteria. A total of 437,722 CpGs remained for analysis.

### Discovery and replication EWAS in Cohort 1

EWAS were separately performed to test the association of each CpG methylation with PEth and AUDIT-C score in the discovery and replication samples. To adjust for significant global confounding factors, we followed a comprehensive analysis pipeline developed by Lehne et al. [51]. Since previous studies have shown that a large number of CpGs were significantly associated with age [52], smoking status [12], race [53], HIV status, and HIV-1 VL [14], these variables were adjusted in the models. The cell proportions of six cell types were also adjusted in the models [54]. The log10 of viral load (log_10_*VL*) and ART adherence were adjusted in the replication sample. In addition, a recent study reported by Jiao et al. [55] demonstrated that sample position affected the measurement of DNAm in Illumina methylation arrays and may introduce biases and increase batch effects. Thus, we adjusted positional effects in the models to further address confounding effects. Epigenome-wide significance was set at a Benjamini–Hochberg false discovery rate (FDR) < 0.05 in the discovery sample. Significance in the replication sample was set at $p\, < \, \frac{{0.05}}{{{\mathrm{number}}\,{\mathrm{of}}\,{\mathrm{CpGs}}\,{\mathrm{being}}\,{\mathrm{tested}}}}$. Analytical models present as the following:

#### First generalized linear model

For discovery,

$$\beta \sim	 \, {\mathrm{ln}}\left( {\mathrm{{PEth}}} \right) + {\mathrm{Position}} + {\mathrm{HIV}}\,{\mathrm{status}} + {\mathrm{Smoker}} + {\mathrm{Race}} \\ 	+ {\mathrm{Age}} + {\mathrm{WBC}} + {\mathrm{CD8T}} + {\mathrm{CD4T}} + {\mathrm{Granulocyte}} \\ 	+ {\mathrm{NK}} + {\mathrm{B}}\,{\mathrm{cell}} + {\mathrm{Monocyte}} + {\mathrm{PC1}}_{{\mathrm{control}} - {\mathrm{probes}}} \\ 	+ \cdot \cdot \cdot + {\mathrm{PC30}}_{{\mathrm{control}} - {\mathrm{probes}}}$$

For replication,

$$\beta \sim 	\, {\mathrm{ln}}\left( {\mathrm{{PEth}}} \right) + {\mathrm{Position}}\,+\,{\mathrm{log}}_{10} {\mathrm{VL}}\, + {\mathrm{ART}}\, {\mathrm{adherence}} \\ 	+ {\mathrm{Smoker}}\,+\,{\mathrm{Race}} + {\mathrm{Age}} + {\mathrm{WBC}} + \,{\mathrm{CD8T}} + {\mathrm{CD4T}}\\ 	+ {\mathrm{Granulocyte}} + {\mathrm{NK}} + {\mathrm{B}}\,{\mathrm{cell}} + {\mathrm{Monocyte}} \\ 	+ {\mathrm{PC1}}_{{\mathrm{control}} - {\mathrm{probes}}} + \cdots + {\mathrm{PC30}}_{{\mathrm{control}} - {\mathrm{probes}}}.$$

#### Principal component analysis (PCA) of intermediary residuals

We then performed a PCA on the resulting regression residuals. The top five principal components (PCs) on the residuals (*PC*1_residuals_,…,*PC*5_residuals_) were adjusted in the final model.

#### A final generalized linear model for identifying differential methylation

We performed a final generalized linear regression analysis for each CpG predicting the *β* as a function of the In(PEth) adjusted for confounders and the top five residual PCs derived from the model above.

The same models were also used for EWAS on AUDIT-C score in discovery and replication samples, where the independent variable ln(PEth) was replaced by the AUDIT-C. To evaluate whether the residual DNAm was adjusted for confounding effect in the above model, we tested the correlations between the top 30 PCs and position, batch, age, race, smoking status, WBC, and six cell-type proportions in before and after QC, respectively.

### Meta-analysis of EWAS in Cohort 1

An EWAS meta-analysis was conducted by combining the findings from the discovery and the replication stages. For each CpG, we obtained effect size estimates and *p* values from the two samples and weighted the effect size estimates by their estimated standard errors. Then, the summary statistics of the two samples were combined using a sample-size weighted meta-analysis using the METAL program [56]. Epigenome-wide significance was set at an FDR < 0.05.

As a comparison of meta-EWAS findings, we conducted a single EWAS in a total of 1,047 samples combining the discovery sample and replication samples together. The batch effect and positional effect were removed by using ComBat [57]. The analytical models and covariables were the same as described above.

### Polygenic methylation score (PGMS)

We constructed a PGMS for each individual as a weighted sum of the CpG *β* values using the effect size estimated from the EWAS as weights [13]. In detail, the PEth-related CpGs identified in the meta-analysis were chosen to construct the PGMS. Then, the PGMS was applied to establish a prediction model for HAD in Cohort 2.

$$\hat M_i = \mathop {\sum }\limits_{j = 1}^q \hat a_j\beta _{ij},$$

$\hat M_i$: the PGMS of individual *i*;

$\hat a_j$: the estimated coefficient for CpG probe *j*;

*β*_*ij*_: the methylation *β* value for individual *i* at CpG probe *j*.

### Adjusted *R*^2^ and incremental adjusted *R*^2^

We used the adjusted *R*^2^ to estimate the phenotypic variances explained by the DNAm. The adjusted *R*^2^ represented the percentage of variation explained by only the independent variables that affected the dependent variable. Here, the adjusted *R*^2^ was the proportion of the variance of the PEth values, AUDIT-C scores, or AUDIT scores that were explained by the individual CpG or the linear combination of CpGs.

We applied the incremental adjusted *R*^2^ (incremental *R*^2^) as one of the parameters for feature selection as described below. The incremental *R*^2^ was used to determine whether a new predictor increases the predictive ability above and beyond that provided by an existing model. It was calculated for each selected CpG or the linear combination of selected CpGs.

### Feature selection using elastic net regularization (ENR)

CpG features were separately preselected from the EWAS results on PEth and on AUDIT-C in Cohort 1. The selected features were used to evaluate the prediction of HAD. Using the ENR method, we performed 10-fold cross-validation for feature selection in the training sample of Cohort 2. Here, we randomly selected 80% of the samples in Cohort 2 and cross-validated them to obtain the values for the ENR tuning parameters. The following steps were taken to select the CpG features and to evaluate their performance.

Step 1. *Preselection CpGs*. Because DNAm of CpGs under the epigenome-wide significance threshold may collectively account for phenotype variation and may improve prediction of a phenotype, we preselected PEth-associated CpGs with a meta *p* < 1E-04 from the meta-EWAS in Cohort 1 for both PEth and AUDIT-C. The preselected CpGs were used to establish the predictive model in the training set of Cohort 2.

Step 2. *Importance ranking CpGs*. In the training set of Cohort 2, we performed an ENR for feature selection among the preselected CpGs. We extracted the coefficients for the model with the lambda value corresponding to the minimum mean cross-validated error. This procedure was repeated *N* times. We excluded the CpGs with the percentage of zero coefficients larger than 95%. All selected CpGs were ranked according to the summation of the absolute value of the *N* coefficients.

Step 3. *Model building by ENR in the training set*. CpG features were selected based on the area under the receiver operating characteristic curve (AUC) and the incremental *R*^2^ for different numbers of CpG sets. The model with the best performance was determined, and the optimal values of the parameters in the best model were found by performing cross-validation in ENR.

Step 4. *Model performance testing in the testing set*. The performance of the CpG features selected from the training set was evaluated in the testing set using AUC, balanced accuracy, and incremental *R*^2^.

A sensitivity test using different cutoffs of *p* values was performed to select the model with the best performance. Different sets of CpGs with *p* value <1E−06, <1E−05, <1E−4, <1E−3 were selected for feature selection in the training sample and model evaluation in the testing sample. The CpG set with the best performance was determined in the final model.

All analyses were performed using R software (https://www.r-project.org/). ENR was performed using the function “cv.glmnet” in the “glmnet” package.

### Biological interpretation of the prediction model

Gene enrichment analysis was performed using the CpGs from the final prediction model to understand the underlying biological significance. We applied the web-accessible, gene annotation term-based Database for Annotation, Visualization and Integrated Discovery (DAVID) for gene enrichment analysis (http://david.niaid.nih.gov) [58]. The expanded DAVID Knowledgebase integrates almost all major and well-known public bioinformatics resources [59]. A significant pathway was set as an FDR < 0.05.

## Results

### EWAS identifies new DNA methylation CpGs for PEth but not for self-reported alcohol consumption

Two analyses of EWAS on PEth values and on AUDIT-C scores were separately conducted in Cohort 1. Phenotypically, as expected, PEth level and AUDIT-C score were highly correlated (*r* = 0.45, *p* < 2.00E−16) (Supplementary Fig. S1a). Compared with the non-HAD group, the HAD group had a greater AUDIT-C score (*p* = 5.42E−132) and a higher level of PEth (*p* = 3.47E−33) (Supplementary Fig. S1b). Demographic and clinical variables are presented in Table 1.

#### Discovery EWAS on PEth and on AUDIT-C

Prior to data QC, we found 10 PCs out of 30 PCs in DNAm was significantly correlated with position and batch effect, 4 PCs correlated with WBC, 2 PCs correlated with CD8T, 1 PC correlated with CD4T, and 2 PCs correlated with monocyte ($p \, < \, \frac{{0.05}}{{30}} = 1.67E - 03$) (Supplementary Fig. S2a). After adjusted confounding effects in the model, residual methylation showed no correlations with position, batch, age, race, smoking status, WBC, or six cell-type proportions (Supplementary Fig. S2b), suggesting that the EWAS findings below are unlikely contributed by non-specific variables in the cohort.

We identified 11 epigenome-wide significant CpGs on PEth (FDR = 4.14E−04~3.50E−02) (Supplementary Fig. S3a, Table S2). The EWAS analysis showed minimal inflation (*λ* = 1.086) (Supplementary Fig. S3b). The 11 significant CpGs were located on 11 genes: *SLC7A11* (solute carrier family 7 member 11), *DYRK2* (dual specificity tyrosine phosphorylation regulated kinase 2), *SLC43A1* (solute carrier family 43 member 1), *CCDC71* (coiled-coil domain containing 71), *ABAT* (4-aminobutyrate aminotransferase), *FOXP1* (forkhead box P1), *WDR1* (WD repeat domain 1), *FBLN2* (Fibulin 2), *LOC221710*, *HERV-FRD*, and *C1orf161*. Seven of 11 CpGs were negatively associated with PEth while 4 of 11 were positively associated with PEth.

We found no CpGs that reached an epigenome-wide significance threshold for self-reported AUDIT-C scores. Three of the 11 CpGs associated with PEth showed association with AUDIT-C ($p \, < \, \frac{{0.05}}{{11}} = 4.55E -03$): cg13442969 (*DYRK2*) (*p* = 1.78E−03), cg11376147 (*SLC43A1*) (*p* = 2.81E−03), and cg25221975 (*FBLN2*) (*p* = 1.96E−03). It is noteworthy that all 11 CpGs associated with PEth showed the same direction as the associations with the AUDIT-C scores in the discovery set.

#### Replication EWAS on PEth and on AUDIT-C

In the replication sample, we found one epigenome-wide significant CpG associated with PEth: cg20414364 (*LOC728613*) (Supplementary Fig. S4). For the 11 PEth-associated CpGs identified in the discovery sample, nine CpGs were overlapped between discovery and replication samples. We found that four out of nine CpGs showed significance for PEth ($p \, < \, \frac{{0.05}}{9} = 5.56E - 03$), although they did not reach epigenome-wide significance (*p* ranged from 1.00E−05 to 2.56E−03) (Supplementary Table S2). The four CpGs were located on three genes: cg17962756, cg13442969 (*DYRK2*), cg11376147 (*SLC43A1*), and cg26689780 (*WDR1*).

As expected, the analysis of the EWAS on AUDIT-C scores revealed no CpG reaching epigenome-wide significance in the replication sample. Only one of nine CpGs associated with PEth were associated with AUDIT-C score ($p \, < \, \frac{{0.05}}{9} = 5.56E - 03$) (cg11376147 in *SLC43A1* with *p* = 2.74E−04) and showed the same direction as the association of PEth.

#### Meta-analysis

**Fig. 2:**
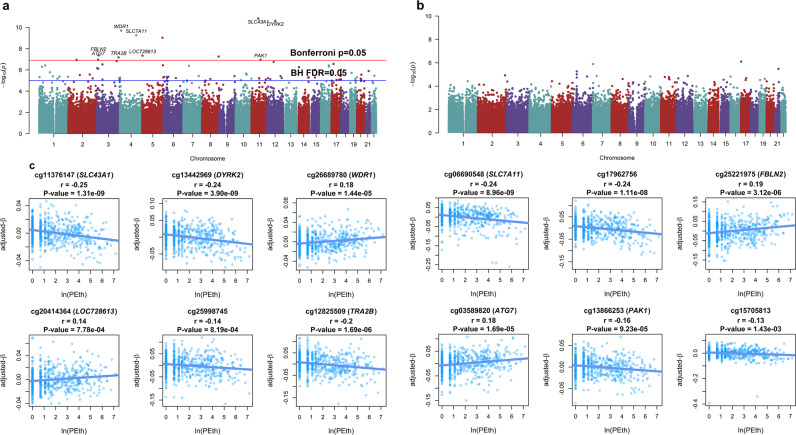
Meta-analyses of epigenome-wide association studies of alcohol consumption (blue line: Benjamini–Hochberg false discovery rate (FDR) cutoff; red line: Bonferroni correction cutoff). **a** Manhattan plot of chromosomal locations of −log_10_ (*p*) for the association between the natural logarithm of phosphatidylethanol (ln(PEth)) and 408,583 CpGs in the meta-analysis. **b** Manhattan plot of chromosomal locations of −log_10_ (*p*) for the association between Alcohol Use Disorders Identification Test-Consumption (AUDIT-C, first three items of AUDIT) and 408,583 CpGs in the meta-analysis. **c** Scatterplots of the adjusted *β* values (adjust confounding factors and use residuals of *β* values) of the 12 Bonferroni significant CpGs (CpGs above the red line in **a**) against the ln(PEth) value. All 12 CpGs were significantly correlated with ln(PEth) with $p < \frac{{0.05}}{{12}} = 4.17E - 03$.

A meta-analysis revealed 83 epigenome-wide significant CpGs on PEth (FDR = 4.94E−06 ~ 4.97E−02) (Table 2 and Fig. 2a). Of note, despite removing batch effects and position effects, a single EWAS conducted in the combining the discovery and replication samples revealed a greater number of *λ* than meta- EWAS (1.442 for the EWAS for combining samples and 1.130 for meta-EWAS) (Supplementary Fig. S5), suggesting that meta-EWAS was less likely inflated and biased than the single EWAS.

**Table 2:** Significant epigenome-wide DNA methylation CpGs associated with phosphatidylethanol (PEth) in the meta-analysis of Cohort 1.

Probe		CHR	Position	Gene	Group	Incremental adjusted R2	Discovery		Replication		Meta-analysis			Reference
							*t*	*P* value	*t*	*P* value	*Z* score	*P* value	FDR	
1	cg11376147^a^	11	57261198	*SLC43A1*	Body	4.49%	−5.18	3.28E−07	−4.48	1.00E−05	−6.75	1.46E−11	4.94E−06	[28, 29]
2	cg13442969	12	68044208	*DYRK2*	5UTR	4.87%	−5.40	1.01E−07	−4.10	4.97E−05	−6.68	2.42E−11	4.94E−06	[29]
3	cg26689780	4	10079554	*WDR1*	Body	4.20%	5.08	5.36E−07	3.98	8.39E−05	6.36	1.99E−10	2.71E−05	[28]
4	cg06690548	4	139162808	*SLC7A11*	Body	6.40%	−6.24	9.47E−10	−2.45	1.48E−02	−6.21	5.46E−10	5.57E−05	[28, 29]
5	cg17962756	5	172769199	*NA*	NA	5.25%	−5.58	3.98E−08	−3.04	2.56E−03	−6.12	9.55E−10	7.81E−05	[28, 29]
6	cg25221975^a^	3	13663444	*FBLN2*	Body	4.27%	5.07	5.58E−07	2.62	9.25E−03	5.48	4.29E−08	2.66E−03	[29]
7	cg20414364	5	1608614	*LOC728613*	Body	0.87%	2.46	1.41E−02	5.61	3.99E−08	5.47	4.56E−08	2.66E−03	
8	cg25998745^a^	8	142028625	*NA*	NA	2.33%	−3.81	1.59E−04	−3.98	8.30E−05	−5.43	5.49E−08	2.80E−03	[29, 65]
9	cg12825509	3	185648568	*TRA2B*	Body	3.52%	−4.61	5.10E−06	−3.01	2.77E−03	−5.40	6.62E−08	3.00E−03	[29, 37]
10	cg03589820	3	11585825	*ATG7*	Body	3.34%	4.50	8.46E−06	3.00	2.92E−03	5.31	1.10E−07	3.87E−03	
11	cg13866253	11	77093001	*PAK1*	Body	2.02%	−3.45	6.13E−04	−4.19	3.46E−05	−5.31	1.12E−07	3.87E−03	
12	cg15705813^a^	2	70297499	*NA*	NA	1.78%	−4.13	4.27E−05	−3.40	7.33E−04	−5.30	1.14E−07	3.87E−03	[28, 29]
13	cg19825437^a^	3	169383292	*NA*	NA	3.64%	−4.69	3.45E−06	−2.68	7.58E−03	−5.25	1.52E−07	4.78E−03	[28, 29]
14	cg02583484^a^	12	54677008	*HNRNPA1; HNRPA1L-2*	Body	2.34%	−3.78	1.74E−04	−3.67	2.74E−04	−5.22	1.77E−07	5.17E−03	[28, 29, 65]
15	cg27376514	17	17058422	*MPRIP*	Body	2.64%	4.03	6.48E−05	3.26	1.20E−03	5.14	2.77E−07	7.53E−03	
16	cg18590502	3	49203081	*CCDC71*	5UTR	4.46%	−5.13	4.08E−07	−2.00	4.65E−02	−5.12	3.06E−07	7.82E−03	[29]
17	cg19731612	5	176559334	*NSD1*	TSS1500	1.84%	−3.37	7.97E−04	−3.93	1.02E−04	−5.08	3.69E−07	8.18E−03	[28, 29]
18	cg02256576	16	66995192	*CES3*	5UTR	2.61%	−3.98	7.93E−05	−3.23	1.36E−03	−5.08	3.80E−07	8.18E−03	[28, 29]
19	cg23090529^a^	1	51442133	*NA*	NA	2.64%	−4.03	6.51E−05	−3.17	1.64E−03	−5.08	3.81E−07	8.18E−03	[29, 65]
20	cg25983901	7	46972700	*NA*	NA	2.94%	−4.18	3.45E−05	−2.98	3.08E−03	−5.07	4.09E−07	8.35E−03	[28]
21	cg00944421	16	68269483	*ESRP2*	Body	3.19%	−4.43	1.14E−05	−2.65	8.51E−03	−5.03	4.81E−07	9.35E−03	
22	cg07167185	1	24120017	*LYPLA2*	Body	1.19%	2.82	5.01E−03	4.47	1.01E−05	5.02	5.11E−07	9.50E−03	
23	cg13548452	14	22573606	*NA*	NA	2.44%	−3.82	1.51E−04	−3.30	1.05E−03	−5.01	5.46E−07	9.69E−03	
24	cg00294109	3	3219781	*CRBN*	Body	1.89%	3.43	6.44E−04	3.67	2.76E−04	4.96	6.91E−07	1.18E−02	
25	cg23482898^a^	3	12858887	*CAND2*	Body	2.04%	3.50	5.04E−04	3.56	4.10E−04	4.95	7.60E−07	1.24E−02	[29]
26	cg23028286	15	51614521	*CYP19A1*	5UTR	3.40%	−4.52	7.61E−06	−2.33	2.03E−02	−4.89	9.85E−07	1.50E−02	
27	cg19869698	17	80058686	*NA*	NA	3.88%	4.73	2.86E−06	2.09	3.70E−02	4.89	9.91E−07	1.50E−02	
28	cg11704631	21	36395663	*RUNX1*	Body	2.96%	−4.16	3.66E−05	−2.65	8.40E−03	−4.84	1.29E−06	1.89E−02	[29]
29	cg06983052	1	90288099	*LRRC8D*	5UTR	2.37%	−3.78	1.73E−04	−3.00	2.90E−03	−4.79	1.69E−06	2.33E−02	[28, 29]
30	cg01425762	16	81666633	*CMIP*	Body	0.87%	2.44	1.49E−02	4.54	7.50E−06	4.79	1.71E−06	2.33E−02	
31	cg24135793	19	13122567	*NFIX*	Body	2.00%	−3.49	5.18E−04	−3.29	1.09E−03	−4.76	1.90E−06	2.51E−02	[29, 37]
32	cg00220102	16	8806756	*ABAT*	TSS200	4.26%	−5.11	4.65E−07	−1.47	1.44E−01	−4.75	2.02E−06	2.58E−02	
33	cg24351003	10	88013210	*GRID1*	Body	1.97%	−3.46	5.90E−04	−3.28	1.12E−03	−4.73	2.22E−06	2.75E−02	
34	cg22274745	2	182451537	*CERKL*	Body	1.95%	3.51	4.88E−04	3.16	1.71E−03	4.69	2.73E−06	3.12E−02	
35	cg08250921	16	88111009	*NA*	NA	3.20%	4.42	1.19E−05	2.13	3.42E−02	4.69	2.76E−06	3.12E−02	
36	cg24238409	10	93998677	*CPEB3*	Body	2.92%	4.26	2.43E−05	2.30	2.18E−02	4.68	2.79E−06	3.12E−02	
37	cg13610455	20	37054900	*LOC388796; SNORA71B*	TSS1500	1.59%	3.16	1.65E−03	3.54	4.46E−04	4.68	2.85E−06	3.12E−02	
38	cg10891521	17	81047941	*METRNL*	Body	1.04%	2.63	8.80E−03	4.15	4.08E−05	4.68	2.93E−06	3.12E−02	
39	cg14817906	2	97466833	*CNNM4*	Body	2.66%	4.05	5.88E−05	2.52	1.22E−02	4.67	2.97E−06	3.12E−02	
40	cg24136754	22	37403978	*C22orf33*	TSS200	1.84%	3.40	7.19E−04	3.20	1.49E−03	4.64	3.53E−06	3.60E−02	
41	cg06925984	17	77767242	*NA*	NA	1.90%	−3.45	5.97E−04	−3.12	1.92E−03	−4.63	3.72E−06	3.61E−02	
42	cg08616943	7	130552600	*NA*	NA	1.99%	−3.50	5.14E−04	−3.07	2.28E−03	−4.62	3.78E−06	3.61E−02	
43	cg14395885	9	130700923	*DPM2*	TSS200	0.73%	−2.23	2.62E−02	−4.53	7.91E−06	−4.62	3.87E−06	3.61E−02	
44	cg15033653	12	113587581	*CCDC42B*	TSS200	3.14%	4.41	1.26E−05	2.03	4.29E−02	4.62	3.89E−06	3.61E−02	
45	cg20970380	1	116676103	*C1orf161*	3UTR	4.05%	−4.98	8.80E−07	−1.39	1.66E−01	−4.61	4.06E−06	3.68E−02	
46	cg03044573^a^	1	173835265	*GAS5; SNORD78; SNORD44; SNORD80; SNORD79*	TSS1500	0.97%	−2.98	3.05E−03	−3.63	3.19E−04	−4.60	4.20E−06	3.73E−02	[29]
47	cg23598378	6	42072986	*C6orf132*	Body	1.16%	2.77	5.85E−03	3.86	1.34E−04	4.59	4.41E−06	3.77E−02	[28]
48	cg06059663	1	245319431	*KIF26B*	Body	2.12%	−3.65	2.91E−04	−2.85	4.67E−03	−4.59	4.45E−06	3.77E−02	
49	cg11302401	6	6688847	*NA*	NA	2.63%	−3.97	8.12E−05	−2.47	1.38E−02	−4.59	4.52E−06	3.77E−02	
50	cg17840178	6	30709803	*FLOT1*	Body	3.16%	−4.38	1.42E−05	−2.01	4.55E−02	−4.58	4.62E−06	3.78E−02	
51	cg13966547	1	2406284	*PLCH2*	TSS1500	1.21%	−2.83	4.84E−03	−3.76	1.97E−04	−4.57	4.79E−06	3.82E−02	
52	cg27477373	19	56879645	*ZNF542*	TSS200	1.81%	−3.35	8.71E−04	−3.15	1.74E−03	−4.57	4.93E−06	3.82E−02	
53	cg06937549	5	179046350	*HNRNPH1*	Body	2.33%	−3.76	1.87E−04	−2.67	7.80E−03	−4.56	5.05E−06	3.82E−02	
54	cg26340050	14	105771879	*NA*	NA	3.41%	4.54	7.13E−06	1.81	7.18E−02	4.56	5.08E−06	3.82E−02	
55	cg05303280	15	51632611	*GLDN*	TSS1500	3.46%	−4.55	6.73E−06	−1.78	7.55E−02	−4.56	5.22E−06	3.82E−02	
56	cg21550372	14	100908908	*WDR25*	Body	2.39%	−3.79	1.68E−04	−2.63	8.90E−03	−4.55	5.28E−06	3.82E−02	
57	cg03840289	4	2262318	*MXD4*	Body	1.72%	3.25	1.25E−03	3.25	1.27E−03	4.55	5.33E−06	3.82E−02	
58	cg17521665	6	106546704	*PRDM1*	TSS200	1.32%	−2.92	3.60E−03	−3.59	3.76E−04	−4.53	5.78E−06	3.90E−02	
59	cg10692140	6	30496072	*NA*	NA	1.34%	−2.89	4.07E−03	−3.63	3.21E−04	−4.53	5.83E−06	3.90E−02	
60	cg11599718	12	123357128	*VPS37B*	Body	1.29%	2.89	4.05E−03	3.63	3.27E−04	4.53	5.89E−06	3.90E−02	
61	cg00166216	3	194407860	*FAM43A*	1stExon	2.20%	−3.69	2.44E−04	−2.70	7.21E−03	−4.53	5.91E−06	3.90E−02	
62	cg23019886	12	6277045	*NA*	NA	2.22%	3.68	2.58E−04	2.72	6.87E−03	4.53	5.92E−06	3.90E−02	
63	cg17953300	11	65418265	*SIPA1*	3UTR	1.52%	3.14	1.78E−03	3.33	9.63E−04	4.53	6.01E−06	3.90E−02	
64	cg10440877	2	208378475	*NA*	NA	1.52%	−3.04	2.50E−03	−3.43	6.71E−04	−4.52	6.30E−06	3.98E−02	
65	cg09801924	11	65425948	*RELA*	Body	1.23%	2.77	5.90E−03	3.73	2.17E−04	4.51	6.51E−06	3.98E−02	
66	cg19939130	1	158978468	*IFI16*	TSS1500	2.21%	−3.69	2.51E−04	−2.68	7.72E−03	−4.51	6.52E−06	3.98E−02	
67	cg14259466	10	135090997	*ADAM8*	TSS1500	1.46%	3.06	2.31E−03	3.39	7.76E−04	4.51	6.56E−06	3.98E−02	
68	cg11846968	20	31823545	*PLUNC*	TSS1500	0.84%	−2.36	1.89E−02	−4.21	3.25E−05	−4.51	6.62E−06	3.98E−02	
69	cg18568145	1	155225764	*FAM189B*	TSS1500	2.77%	−4.11	4.67E−05	−2.19	2.88E−02	−4.50	6.73E−06	3.99E−02	
70	cg20699548	8	71060638	*NCOA2*	Body	2.71%	−4.05	5.90E−05	−2.24	2.57E−02	−4.49	7.12E−06	4.15E−02	
71	cg22537604	19	43857074	*CD177*	TSS1500	2.08%	−4.31	1.92E−05	−1.93	5.37E−02	−4.49	7.31E−06	4.19E−02	
72	cg23747342	12	25539794	*NA*	NA	0.99%	2.60	9.70E−03	3.89	1.19E−04	4.48	7.37E−06	4.19E−02	
73	cg21845080	3	196065306	*TM4SF19*	TSS200	0.44%	1.84	6.65E−02	4.76	2.77E−06	4.47	7.76E−06	4.29E−02	
74	cg14728380	17	80280330	*SECTM1*	Body	2.44%	3.81	1.55E−04	2.48	1.36E−02	4.47	7.79E−06	4.29E−02	
75	cg16423756	11	122526190	*UBASH3B*	TSS1500	3.16%	4.36	1.60E−05	1.86	6.31E−02	4.47	7.89E−06	4.29E−02	
76	cg20732160	3	48590040	*PFKFB4*	Body	2.58%	−3.99	7.58E−05	−2.26	2.41E−02	−4.46	8.13E−06	4.29E−02	
77	cg00970435	17	66380327	*ARSG*	Body	1.11%	−2.67	7.82E−03	−3.77	1.90E−04	−4.46	8.16E−06	4.29E−02	
78	cg09191335	20	35241157	*SLA2*	3UTR	2.24%	3.66	2.77E−04	2.63	8.81E−03	4.46	8.18E−06	4.29E−02	
79	cg15690475	17	44101453	*MAPT*	Body	1.86%	3.38	7.68E−04	2.92	3.68E−03	4.44	8.82E−06	4.55E−02	
80	cg27155460	10	45420821	*TMEM72*	Body	2.70%	4.03	6.52E−05	2.19	2.89E−02	4.44	8.91E−06	4.55E−02	
81	cg09635954	7	29605624	*PRR15*	5UTR	0.80%	−2.35	1.94E−02	−4.08	5.39E−05	−4.42	9.82E−06	4.95E−02	
82	cg03394159	8	29197844	*DUSP4*	Body	1.90%	3.32	9.79E−04	2.96	3.31E−03	4.42	1.01E−05	4.97E−02	
83	cg21366673	6	30459512	*HLA-E*	Body	3.23%	−4.38	1.46E−05	−1.76	7.92E−02	−4.42	1.01E−05	4.97E−02	[28]

FDR: Benjamini–Hochberg false discovery rate; The CpGs in the table are significant (FDR < 5.00E−02) in meta-analysis.

^a^The CpGs that are significant using African Ancestry sample in Liu et al. [29].

A majority of these CpGs (66 out of 83 CpGs) were in a gene region, including 18 CpGs in the promoter, 1 CpGs in the first exon, and 9 CpGs in the UTR regions. With a stringent significant threshold, 12 CpGs showed a Bonferroni adjusted *p* < 5.00E−02. These 12 CpGs mapped to 9 genes, including 3 novel genes (*LOC728613, ATG7*, and *PAK1*) for alcohol consumption and 6 genes (*SLC43A1, DYRK2*, *WDR1*, *SLC7A11*, *FBLN2*, and *TRA2B)* previously reported to be related to alcohol consumption [28, 29, 37].

Interestingly, even with an increased sample size in the meta-analysis, we found no epigenome-wide significant CpG site of the meta-EWAS on AUDIT-C scores (Fig. 2b).

We further tested the correlation between the *β* values of the 12 CpGs with Bonferroni significance and PEth. All 12 CpGs were significantly correlated with PEth levels with $p \, < \, \frac{{0.05}}{{12}} = 4.17E - 03$ after the model was adjusted for confounding factors (Fig. 2c), 4 of the 12 CpGs were positively correlated with PEth, and the remaining 8 CpGs were negatively correlated with PEth.

### PEth-associated CpG sites improve the prediction of HAD in Cohort 1

Because PEth itself was highly correlated with AUDIT-C scores and differed significantly between the HAD and the non-HAD groups, we were interested in whether PEth-associated CpG DNAm improved the prediction of HAD compared with the prediction of HAD using PEth alone. We found that the AUC was 74.2% for PEth alone, 76.8% with the 12 Bonferroni significant CpGs and PEth, and 87.2% with the 83 epigenome-wide significant CpGs and PEth (Supplementary Fig. S6). Thus, DNAm features improved the prediction of hazardous alcohol consumption compared with PEth alone in the same cohort.

### PGMS derived from 83 PEth-associated CpGs is correlated with alcohol consumption in an independent sample

To be consistent with the analysis in Cohort 1, we performed an EWAS on AUDIT-C score in Cohort 2. We found no epigenome-wide significant CpG for AUDIT-C. An EWAS for a full scale of AUDIT score also revealed no significant CpG.

A PGMS constructed from the 83 PEth-associated CpGs was highly correlated with the self-reported 10-item AUDIT score in Cohort 2 (*r* = 0.40, *p* = 5.47E−19). The incremental *R*^2^ of the association between the PGMS corresponding to 83 PEth-related CpGs and the 10-item AUDIT score was 0.0976, which implied that the PGMS explained 9.8% of the variance of the full AUDIT score in an independent population (Supplementary Fig. S7a).

We further tested whether the PGMS derived from the PEth-associated CpGs was separately correlated with self-reported alcohol consumption (AUDIT-C, first three items of AUDIT) and self-reported problem alcohol drinking behaviors (AUDIT-P, item 4–10 of full AUDIT). We found that the PGMS was significantly correlated with AUDIT-C score (*r* = 0.36, *p* = 3.36E−15) (Supplementary Fig. S7b) and AUDIT-P score (*r* = 0.34, *p* = 1.29E−10) (Supplementary Fig. S7c). The correlation of the PGMS was slightly stronger with the AUDIT-C score than with the AUDIT-P score.

### PEth-associated DNA methylation CpG sites predict HAD in Cohort 2

We found no statistically significant difference in the characteristics between the training set and the testing set in Cohort 2 (Supplementary Table S3). As shown in Supplementary Fig. S8, a sensitive test showed that the best performance model was a panel of CpGs preselected at *p* < 1E−04 assessed by AUC and incremental *R*^2^. Of note, although a larger cutoff value, e.g., *p* < 1E−03, showed a greater incremental *R*^2^, the AUC was less than the CpG set at the cutoff of *p* < 1E−04, which may be due to the increased background noise with a larger number of preselected CpGs at *p* < 1E−03. Therefore, the panel of CpGs with *p* < 1E−04 from the meta-EWAS in Cohort 1 were preselected for feature selection.

**Fig. 3:**
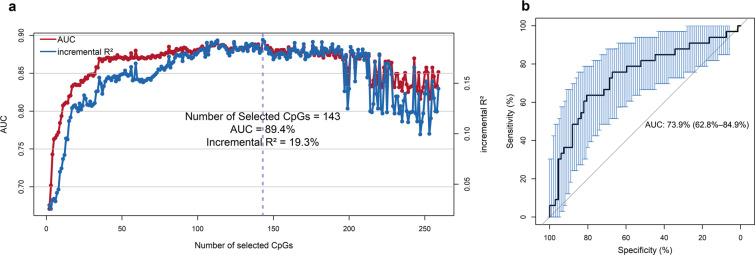
Feature selection using elastic net regularization (ENR) for hazardous alcohol drinking (HAD). **a** The area under the receiver operating characteristic curve (AUC) and the incremental adjusted *R*^2^ (incremental *R*^2^) of the selected CpGs using the ENR method. A set of CpGs associated with the natural logarithm of phosphatidylethanol (In(PEth)) in cohort 1 (*p* < 1.00E−04) was preselected for ENR analysis in training samples of Cohort 2. Incremental *R*^2^ denotes the difference in adjusted *R*^2^ between the model with the predicted variable and the model without the predicted variable. **b** The ROC curve for HAD prediction in the testing set of Cohort 2 using the 143 ENR-selected CpGs from the training samples.

A total of 259 CpGs were preselected to build a predictive model in the training set of Cohort 2. All 259 CpGs were ranked according to the summation of the absolute value of the *N* coefficients. As shown in Fig. 3a, a panel of 143 CpGs (Supplementary Table S4) showed the greatest AUC with 89.4% and the highest incremental *R*^2^ with 19.3% in the training set. Therefore, a model of 143 CpGs was validated in the testing set.

In the testing set, we found that the 143 CpGs model showed an AUC of 73.9%, a balanced accuracy of 62.3%, and an incremental *R*^2^ of 5.9% (Fig. 3b). The results show that the 143 selected PEth-associated CpGs enabled the good prediction of HAD. Notably, the panel of 143 CpGs included 44 epigenome-wide significant CpGs for meta-EWAS on PEth in Cohort 1.

Using the same approach for the analysis of feature selection of AUDIT-C-associated CpGs from Cohort 1 to predict HAD in Cohort 2, a panel of 18 CpGs were selected from 54 CpGs with *p* < 1E−04. In the training set, the AUC was 70.2%, and the incremental *R*^2^ was 2.2%. In the testing set, the AUC was 57.6% (46.1–69.1%), and the incremental *R*^2^ was 1.1%.

### Biological interpretation of the 143 identified PEth-associated CpGs

The 143 CpGs from the final predictive model were annotated on 117 genes. Gene enrichment analysis yielded one significant annotation terms GO:0048519~negative regulation of biological process (BP) (*p* = 3.00E−06; FDR = 5.39E−03). Besides this significant pathway, the top 14 pathways (11 BP; 1 molecular function (MF); 2 cellular components (CC)) with *p* < 1E−03, an arbitrarily cutoff, was presented in Supplementary Fig. S9. The 11 BP pathways included GO:0048519~negative regulation of BP**;** GO:0030155~regulation of cell adhesion (*p* = 5.86E−05); GO:0048523~negative regulation of cellular process (*p* = 1.08E-04); GO:0044707~single-multicellular organism process (*p* = 1.36E−04); GO:0051240~positive regulation of multicellular organismal process (*p* = 3.14E−04); GO:0007275~multicellular organism development (*p* = 4.24E−04); and GO:0048731~system development (*p* = 5.19E−04); GO:0065009~regulation of MF (*p* = 7.01E−04); GO:0048856~anatomical structure development (*p* = 7.09E−04); GO:1902107~positive regulation of leukocyte differentiation (*p* = 8.43E−04); GO:0048812~neuron projection morphogenesis (*p* = 9.11E−04).The MF pathway is GO:0047485~protein N terminus binding (*p* = 2.51E−04). The two CC pathways are GO:0031974~membrane-enclosed lumen (*p* = 5.70E−04); GO:0043233~organelle lumen (*p* = 9.33E−04).

## Discussion

Using samples from two distinct populations, we have demonstrated that an objective phenotype, PEth, is a robust phenotype for identifying DNAm in blood associated with HAD and that PEth-associated CpGs are predictive of HAD. We revealed 83 CpGs associated with PEth, while none of the CpGs were associated with self-reported alcohol consumption. A PGMS derived from the 83 CpGs explained 9.8% of the variance of alcohol consumption in a demographically and clinically independent sample. We further showed that the 83 CpGs combined with PEth improved 13% of AUC of predicting HAD compared with the AUC of predicting HAD by PEth alone. Importantly, we identified a panel of 143 CpGs that were relevant to PEth levels in a mostly HIV-positive sample and that predicted self-reported HAD in an HIV-negative sample. The 143 CpGs included five CpGs that were previously included in the DNAm biomarker panel for prediction of alcohol consumption and five CpGs were Bonferroni significant associated with alcohol consumption in an African Ancestry sample in Liu et al. (Supplementary Fig. S10) [29]. Interestingly, a panel of CpGs related to self-reported AUDIT-C score showed poor predictive performance for HAD. Together, these findings suggest that PEth-associated DNAm features, but not DNAm for self-reported alcohol consumption, is a robust biomarker in predicting hazardous alcohol consumption that may have potential clinical utility.

Emerging evidence suggests that a set of epigenetic modification markers across different tissues is more stable and reproducible than we previously expected [60]. In this study, we replicated 24 CpGs that had previously reported associations with alcohol consumption or alcohol use disorders (Supplementary Table S4). For example, three promoter CpGs, cg19731612 on *NSD1* [28, 29], cg03523740 on *TXLNA* [28, 29], cg18121224 on *NSD1* [28], and cg00407659 on *ANXA6* [28] that were associated with alcohol consumption in previous studies were also significantly associated with PEth in our study. In addition, we revealed multiple new PEth-associated CpGs that are located on the genes involved in tyrosine autophosphorylation, catalyzed phosphorylation of histones H3 and H2B (*DYRK2*) and the serine/threonine p21-activating kinases (*PAK1*), sequence-specific serine/arginine splicing factor (*TRA2B*) functions, and extracellular matrix protein (*FBLN2*). These results suggest that alcohol consumption alters DNAm on the genes involved in the cellular process and epigenetic programming. One intriguing question is whether the significant CpGs for HAD detected from the blood sample is relevant to methylation alteration by alcohol consumption in the brain. Several studies have reported associations of methylation of CpGs with alcohol consumption in the human postmortem brain samples. Several of those CpGs showed nominal significance in our blood samples. For example, cg18362496 in *H19* that was previously reported hypermethylation in AUD brain samples [61] showed a positive association with PEth in our blood sample (*p* = 0.03). A stress-related gene, *KCNK6*, that was previously associated with AUD in brain [31] was nominal significant in the same direction in our study (*p* = 0.04). However, the majority of significant CpGs for alcohol consumption differs between brain and blood samples. The discrepancy is not unexpected considering distinct methylome architectures between the brain and peripheral tissues. Although the findings do not elucidate the etiology of alcohol drinking behavior in brain, our results suggest a peripheral mechanism of how alcohol consumption changes the epigenome, which may lead to medical disorders. Given the inaccessibility of brain tissues in living humans, biomarkers from peripheral cells could be of benefit in the clinical care of HAD patients.

The 83 PEth-associated CpGs identified in a mostly HIV-positive population collectively explained 9.8% of the variance of HAD in an HIV-negative population, suggesting the stability of the DNAm effect of alcohol exposure. Notably, the 9.8% effect size of the PGMS in our study is comparable with the previously reported 12–13.8% effect size of a PGMS in a study with a larger sample size (*N* = 13,317) than our study [29]. We further showed that PGMS was not only significantly associated with recent alcohol consumption (AUDIT-C) (*r* = 0.36, *p* = 3.36E−15) but was strongly associated with the problematic consequences of alcohol use (AUDIT-P) (*r* = 0.34, *p* = 1.29E−10), further indicating that DNAm is a relatively stable marker for the long-term effects of alcohol consumption.

The reproducible CpGs suggest a robust, consistent epigenetic response to alcohol consumption that can serve as biomarkers for clinical use. Using a machine learning approach, we identified a set of 143 CpGs that enables the distinction of HAD and non-HAD individuals. One of the common challenges for machine learning prediction is model overfitting. We took several steps to address this concern: (1) feature preselection and selection were conducted in two different cohorts; (2) the processes of feature selection and model evaluation were carried out in the same cohort but in different sets without overlapping samples; and (3) we applied a newly developed machine learning ENR method to select features in a combination of 10-fold cross-validation. Compared with two traditional penalized regression methods, Ridge [62] and the least absolute shrinkage and selection operator (LASSO) [63], ENR has the advantage of selecting informative features without compromising predictive accuracy and has been shown to outperform both the Ridge and LASSO methods [64]. With these strengths of the analytical approach, we showed that a panel of 143 CpGs performed fairly well in the testing sample set.

Compared with the findings from the largest DNAm biomarker study for alcohol consumption up to date by Liu et al. [29], we found that a small proportion of CpGs is indeed overlapping between two studies. Despite many differences between ours and Liu’s studies, e.g., sample size, phenotype assessment, DNAm profiling array, and analytical strategy, nine epigenome-wide significant CpGs are identical between ours and Liu et al.’s studies in African Americans. These nine overlapped CpGs are located in five genes: *SLC43A1, FBLN2, HNRNPA1, CAND2*, and *GAS5*. Five biomarker-CpGs are overlapping between the two studies. The overlapped CpGs are located on *SLC7A11, DYRK2, TRA2B, NCOA2*, and *GPR133*. Enrichment analysis suggests the overlapped CpGs are not discovered by chance across the two studies ($T_{\chi ^2}$= 2400 and *p* = 0 for epigenome-wide significant CpGs; $T_{\chi ^2}$ = 486.4 and *p* = 0 for biomarker-CpGs). Therefore, the overlapped CpGs across two very different studies further underscore the stability and reproducibility of DNAm as a biomarker for alcohol consumption.

Several limitations should be considered in interpreting the current findings. (1) There was a lack of power to detect sex-specific associations between CpGs and HAD. It is well known that HAD in men and women is epidemiologically and mechanistically different. The individuals in Cohort 1 were all men and ~50% of the individuals in Cohort 2 were women. These samples are insufficient to seek sex-specific DNAm markers. (2) The DNAm signatures were identified from whole blood samples that lacked cell-type-specific profiles. Future analyses using cell-type-specific CpGs may improve prediction performance. (3) The 143 CpGs in the DNAm signature were preselected from an HIV-positive sample, while the prediction model was built and validated in an HIV-negative sample. We expect to improve the predictive efficiency in a relatively homogenous sample in future studies. (4) Other psychiatric disorders such as depression are common in HAD, which might have confounded the findings. Validation of the prediction panel on other alcohol use-related phenotypes, e.g., alcohol use disorder, and address other psychiatric disorders are necessary to confidently claim the predictive performance and accuracy for clinical use.

In summary, to the best of our knowledge, this is the first study to demonstrate that PEth is a robust phenotype for detecting subtle DNAm changes associated with alcohol consumption compared with self-reported alcohol use data. PEth-associated DNAm markers predicted HAD with a good accuracy. These findings suggest that DNAm signatures may have clinical utility as biomarkers for alcohol consumption, and further development and testing of these biomarkers are warranted.

## Supplementary information

Supplementary information

## Data Availability

Demographic variables, clinical variables, and methylation status for the VACS samples were submitted to the GEO dataset (GSE117861) and are available to the public. All codes for analysis are also available upon a request to the corresponding author.
