# Supplementary material for: DNA methylation signature on phosphatidylethanol, not on self-reported alcohol consumption, predicts hazardous alcohol consumption in two distinct populations
Source: Mol Psychiatry. 2020 Feb 7;26(6):2238–53. doi: 10.1038/s41380-020-0668-x (PMC8440221; doi:10.1038/s41380-020-0668-x)
Supplement: Supplementary file 1 — Supplementary information [file 41380_2020_668_MOESM1_ESM.docx]

**SUPPLEMENTARY MATERIALS AND METHODS**

**DNA Methylation Signature on Phosphatidylethanol, not Self-Reported Alcohol Consumption, Predicts Hazardous Alcohol Consumption in Two Distinct Populations**

Xiaoyu Liang, Amy C. Justice, Kaku So-Armah, John H. Krystal, Rajita Sinha and Ke Xu

Table of Contents

[**Definition of phenotypes** 2](#_Toc29565149)

[**DNA methylation profile and quality control** 2](#_Toc29565150)

[**Discovery and replication EWAS in Cohort 1** 3](#_Toc29565151)

[**Supplementary Table S1.** Description of alcohol-related phenotypes that were used in our paper 5](#_Toc29565152)

[**Supplementary Table S2.** Significant epigenome-wide DNA methylation CpGs associated with Phosphatidylethanol (PEth) in discovery and replication samples of Cohort 1 6](#_Toc29565153)

[**Supplementary Table S3.** Demographic and clinical characteristics of the samples between training set and testing set in Cohort 2 7](#_Toc29565154)

[**Supplementary Table S4.** Elastic net regularization (ENR) selected a set of 143 CpGs for predicting Hazardous Alcohol Drinking (HAD) 8](#_Toc29565155)

[**Supplementary Fig. S1.** Correlation between the natural logarithm of Phosphatidylethanol (ln(PEth)) and Alcohol Use Disorders Identification Test-Consumption (AUDIT-C, first 3 items of AUDIT) score in Cohort 1 18](#_Toc29565156)

[**Supplementary Fig. S2.** Correlation plots show the correlation between the top 30 principal components on DNA methylation (X-axis) and confounding factors including six cell types (Y-axis) 19](#_Toc29565157)

[**Supplementary Fig. S3.** Manhattan plot and quantile-quantile (QQ) plot for the discovery set of Cohort 1 20](#_Toc29565158)

[**Supplementary Fig. S4.** Manhattan plot and quantile-quantile (QQ) plot for the replication set of Cohort 1 21](#_Toc29565159)

[**Supplementary Fig. S5.** Quantile-Quantile (QQ) plot for meta-analysis and combined analysis 22](#_Toc29565160)

[**Supplementary Fig. S6.** Receiver Operating Characteristic (ROC) curves of Phosphatidylethanol (PEth) with and without DNA methylation for predicting Hazardous Alcohol Drinking (HAD) 23](#_Toc29565161)

[**Supplementary Fig. S7.** Correlation between PolyGenic Methylation Score (PGMS) and alcohol-related phenotypes 24](#_Toc29565162)

[**Supplementary Fig. S8.** Sensitivity test of the preselected CpG set with different cutoff values 25](#_Toc29565163)

[**Supplementary Fig. S9.** Database for Annotation, Visualization and Integrated Discovery (DAVID) pathway analysis for the 143 CpGs selected by elastic net regularization (ENR) with $p<$1E-03 26](#_Toc29565164)

[**Supplementary Fig. S10.** Comparison of the identified CpG sets for alcohol consumption between the present study and Liu et al (2018) 27](#_Toc29565165)

[**References** 28](#_Toc29565166)

# **Definition of phenotypes**

Alcohol consumption was measured by PEth and 3-item AUDIT-C in cohort 1, and 10-item AUDIT in cohort 2. The definition of hazardous alcohol drinking is defined in **Supplementary Table S1**. Of note, no woman subject was included in Cohort 1.

# **DNA methylation profile and quality control**

In Cohort 1, DNAm for the discovery sample was profiled by using the Illumina Infinium HumanMethylation450 Beadchip (Illumina HM450K) (San Diego, CA, USA). DNAm for the replication sample was assessed by using the Illumina Infinium MethylationEPIC Beadchip (Illumina EPIC) (San Diego, CA, USA). In Cohort 2, DNAm was measured by using Illumina HM450K. All samples in Cohorts 1 and 2 were processed at the Yale Center for Genomic Analysis [1].

Methylation raw data were retrieved using the minfi R package (version 1.18.1), and downstream analyses were performed using minfi and R. We performed the probe normalization and batch-correction procedure using the pipeline reported by Lehne *et al.*[2]. We removed CpGs on sex chromosomes and CpGs within 10 base pairs of single nucleotide polymorphisms. In Cohort 1, a total of 437,722 CpGs from 450K array remained in the discovery sample and 846,604 CpGs from EPIC array remained for the replication sample after quality control (QC). A total of 48.26% common CpGs (408,583) were analyzed in meta-analyses. We also compared the predicted sex with self-reported sex. All samples were matched as males. In Cohort 2, we applied the same QC criteria. A total of 437,722 CpGs remained for analysis. Methylation inferred sex matched with self-reported sex data in this cohort.

Six cell types (CD4+ T cells, CD8+ T cells, NK T cells, B cells, monocytes, and granulocytes) in the blood were estimated in each sample for both cohorts using the method described by Houseman *et al*. [3, 4].

# **Discovery and replication EWAS in Cohort 1**

EWAS were separately performed to test the association of each CpG methylation with PEth and AUDIT-C score in the discovery and replication samples. To adjust for significant global confounding factors, we followed a comprehensive analysis pipeline developed by Lehne *et al*. [2]. The primary EWAS model used a DNAm $\beta$-value (the ratio of methylated probe intensity divided by the overall intensity) as the response variable and the continuous natural logarithm of PEth as the predictor variable of interest. Since previous studies have shown that a large number of CpGs were significantly associated with age [5], smoking status [6], race [7], HIV status and HIV-1 VL [1], these variables were adjusted in the models. The cell proportions of 6 cell types were also adjusted in the models. The log10 of viral load ($\log_{10} VL$) and ART adherence were adjusted in the replication sample. In addition, a recent study reported by Jiao *et al*., 2018 [8] demonstrated that sample position affected the measurement of DNAm in Illumina methylation arrays and may introduce biases and increase batch effects. Thus, we adjusted positional effects in the models to further address confounding effects. The same models were also used for EWAS on AUDIT-C score in discovery and replication samples, where the independent variable ln(PEth) was replaced by the AUDIT-C score. Epigenome-wide significance was set at a Benjamini–Hochberg false discovery rate (FDR) < 0.05 in the discovery sample. Significance in the replication sample was set at $p<\frac{0.05}{number of CpGs being tested}$.

1. *First generalized linear model*

We performed a linear model to adjust for the confounders mentioned above in both the discovery and replication models.

For discovery,

$$\beta\sim\ln\left( PEth \right)+Position+HIV status+Smoker+Race+Age+WBC+CD8T+CD4T+Granulocyte+NK+B cell+Monocyte+PC1_{control-probes}+\ldots+PC{30}_{control-probes}$$

For replication,

$$\beta\sim\ln\left( PEth \right)+Position+\log_{10} VL+ART adherence+Smoker+Race+Age+WBC+CD8T+CD4T+Granulocyte+NK+B cell+Monocyte+PC1_{control-probes}+\ldots+PC{30}_{control-probes}$$

1. *Principal component analysis (PCA) of intermediary residuals*

We then performed a PCA on the resulting regression residuals. The top five principal components (PCs) on the residuals ($PC1_{residuals},\ldots,PC5_{residuals}$) were adjusted in the final model.

1. *A final generalized linear model for identifying differential methylation*

We performed a final generalized linear regression analysis for each methylation

marker predicting the $\beta$ as a function of the natural logarithm of the PEth value adjusted for technical and biological factors and the top 5 residual PCs derived from the model above.

| **Supplementary Table S1.** Description of alcohol-related phenotypes that were used in our paper | | | | | | |
| --- | --- | --- | --- | --- | --- | --- |
| **Cohort** | **Phenotype** | **Description** | **HAD** | | **non-HAD** | |
|  |  |  | **Men** | **Women** | **Men** | **Women** |
| 1 | PEth | Phosphatidylethanolis a lipid metabolite of ethanol formed from phosphatidylcholine in erythrocytes | >=20 ng/ml | N/A | <20 ng/ml | N/A |
| 1 | AUDIT-C | Alcohol Use Disorders Identification Test-Consumption items, items 1-3 | >=4 | N/A | <4 | N/A |
| 2 | AUDIT | Alcohol Use Disorders Identification Test, items 1-10 | >=8 | >=7 | <8 | <7 |

| **Supplementary Table S2.** Significant epigenome-wide DNA methylation CpGs associated with Phosphatidylethanol (PEth) in discovery and replication samples of Cohort 1 | | | | | | | | | | | |
| --- | --- | --- | --- | --- | --- | --- | --- | --- | --- | --- | --- |
| **Probe** | | **CHR** | **Position** | **Gene** | **Group** | **Incremental adjusted R2** | **Discovery** | | | **Replication** | |
|  |  |  |  |  |  |  | **t** | **p** | **FDR** | **t** | **p*** |
| 1 | cg06690548 | 4 | 139162808 | *SLC7A11* | Body | 6.40% | -6.24 | 9.47E-10 | 4.14E-04 | -2.45 | 1.48E-02 |
| 2 | cg17962756 | 5 | 172769199 | *NA* | NA | 5.25% | -5.58 | 3.98E-08 | 8.70E-03 | -3.04 | **2.56E-03** |
| 3 | cg13442969 | 12 | 68044208 | *DYRK2* | 5UTR | 4.87% | -5.40 | 1.01E-07 | 1.47E-02 | -4.10 | **4.97E-05** |
| 4 | cg11376147 | 11 | 57261198 | *SLC43A1* | Body | 4.49% | -5.18 | 3.28E-07 | 2.71E-02 | -4.48 | **1.00E-05** |
| 5 | cg18590502 | 3 | 49203081 | *CCDC71* | 5UTR | 4.46% | -5.13 | 4.08E-07 | 2.71E-02 | -2.00 | 4.65E-02 |
| 6 | cg00220102 | 16 | 8806756 | *ABAT* | TSS200 | 4.26% | -5.11 | 4.65E-07 | 2.71E-02 | -1.47 | 1.44E-01 |
| 7 | cg20525486 | 3 | 71111923 | *FOXP1* | Body | 4.37% | 5.11 | 4.71E-07 | 2.71E-02 | NA | NA |
| 8 | cg26689780 | 4 | 10079554 | *WDR1* | Body | 4.20% | 5.08 | 5.36E-07 | 2.71E-02 | 3.98 | **8.39E-05** |
| 9 | cg25221975 | 3 | 13663444 | *FBLN2* | Body | 4.27% | 5.07 | 5.58E-07 | 2.71E-02 | 2.62 | 9.25E-03 |
| 10 | cg04304130 | 6 | 11111894 | *LOC221710; HERV-FRD* | 5UTR | 4.25% | 5.00 | 7.82E-07 | 3.42E-02 | NA | NA |
| 11 | cg20970380 | 1 | 116676103 | *C1orf161* | 3UTR | 4.05% | -4.98 | 8.80E-07 | 3.50E-02 | -1.39 | 1.66E-01 |
| FDR: Benjamini–Hochberg false discovery rate; The CpGs in the table are significant (FDR<5E-02) in discovery of Cohort 1. | | | | | | | | | | | |
| *The significant CpGs in the replication sample (p-value in bold) were defined as p<0.05/9=5.56E-03 (Bonferroni correction). | | | | | | | | | | | |

| **Supplementary Table S3.** Demographic and clinical characteristics of the samples between training set and testing set in Cohort 2 | | |
| --- | --- | --- |
|  | **Training Set** | **Testing Set** |
|  | **(N = 402)** | **(N = 100)** |
| AUDIT | 5.42 ± 5.83 | 6.81 ± 7.12 |
| Age (year) | 28.93 ± 9.01 | 28.72 ± 7.92 |
| Sex (male, %) | 43.28 | 47.00 |
| Race (AA, %) | 18.91 | 21.00 |
| Smoker (%) | 21.64 | 17.00 |
| CD4+ T (%) | 0.18 ± 0.05 | 0.18 ± 0.05 |
| CD8+ T (%) | 0.09 ± 0.04 | 0.09 ± 0.04 |
| NK (%)^a^ | 0.03 ± 0.03 | 0.04 ± 0.04 |
| B cell (%)^a^ | 0.07 ± 0.03 | 0.07 ± 0.02 |
| Monocyte (%)^a^ | 0.08 ± 0.02 | 0.08 ± 0.02 |
| Granulocyte (%)^a^ | 0.59 ± 0.09 | 0.59 ± 0.08 |
| AA: African American, AUDIT: Alcohol Use Disorders Identification Test | | |
| ^a^Cell type compositions estimated by methylation | | |

| **Supplementary Table S4.** Elastic net regularization (ENR) selected a set of 143 CpGs for predicting Hazardous Alcohol Drinking (HAD) | | | | | | | | | | |
| --- | --- | --- | --- | --- | --- | --- | --- | --- | --- | --- |
| **Probe** | | **CHR** | **Position** | **Gene** | **Group** | **Incremental adjusted R2** | **Z-Score** | **P-value** | **FDR** | **Reference** |
| 1 | cg06690548^LA^ | 4 | 139162808 | *SLC7A11* | Body | 6.40% | -6.21 | 5.46E-10 | 5.57E-05 | [9, 10] |
| 2 | cg00716016 | 9 | 3180374 | *NA* | NA | 1.62% | -3.93 | 8.61E-05 | 1.51E-01 |  |
| 3 | cg00103778 | 20 | 30196041 | *NA* | NA | 0.68% | -3.94 | 8.31E-05 | 1.49E-01 |  |
| 4 | cg08688548 | 15 | 83317398 | *CPEB1* | TSS1500 | 2.19% | -3.91 | 9.37E-05 | 1.53E-01 |  |
| 5 | cg09191335 | 20 | 35241157 | *SLA2* | 3UTR | 2.24% | 4.46 | 8.18E-06 | 4.29E-02 |  |
| 6 | cg24713122 | 11 | 46389164 | *DGKZ* | Body | 0.57% | 3.94 | 8.32E-05 | 1.49E-01 |  |
| 7 | cg15049370 | 5 | 149186389 | *PPARGC1B* | Body | 0.85% | 4.19 | 2.77E-05 | 8.45E-02 |  |
| 8 | cg13121938 | 13 | 114871497 | *RASA3* | Body | 1.66% | 4.06 | 4.88E-05 | 1.17E-01 |  |
| 9 | cg25221975 | 3 | 13663444 | *FBLN2* | Body | 4.27% | 5.48 | 4.29E-08 | 2.66E-03 | [9] |
| 10 | cg02256576 | 16 | 66995192 | *CES3* | 5UTR | 2.61% | -5.08 | 3.80E-07 | 8.18E-03 | [9, 10] |
| 11 | cg01883662 | 3 | 196065289 | *TM4SF19* | TSS200 | 0.46% | 4.35 | 1.38E-05 | 5.54E-02 |  |
| 12 | cg07387591 | 20 | 17208649 | *PCSK2* | Body | 0.78% | 3.94 | 8.33E-05 | 1.49E-01 |  |
| 13 | cg01001791 | 22 | 41924687 | *POLR3H; ACO2* | 3UTR | 1.36% | 3.92 | 9.04E-05 | 1.53E-01 |  |
| 14 | cg13706315 | 9 | 134724316 | *NA* | NA | 0.94% | 4.35 | 1.38E-05 | 5.54E-02 | [9] |
| 15 | cg03523740 | 1 | 32645027 | *TXLNA* | TSS1500 | 2.06% | -4.32 | 1.53E-05 | 6.00E-02 | [9, 10] |
| 16 | cg27477373 | 19 | 56879645 | *ZNF542* | TSS200 | 1.81% | -4.57 | 4.93E-06 | 3.82E-02 |  |
| 17 | cg05303280 | 15 | 51632611 | *GLDN* | TSS1500 | 3.46% | -4.56 | 5.22E-06 | 3.82E-02 |  |
| 18 | cg23028286 | 15 | 51614521 | *CYP19A1* | 5UTR | 3.40% | -4.89 | 9.85E-07 | 1.50E-02 |  |
| 19 | cg03458172 | 3 | 157815672 | *SHOX2* | 3UTR | 1.01% | -4.15 | 3.30E-05 | 9.48E-02 |  |
| 20 | cg18076842 | 11 | 93262607 | *C11orf75* | 5UTR | 1.12% | -4.11 | 3.98E-05 | 1.07E-01 |  |
| 21 | cg19825437 | 3 | 169383292 | *NA* | NA | 3.64% | -5.25 | 1.52E-07 | 4.78E-03 | [9, 10] |
| 22 | cg00717678 | 17 | 1554577 | *PRPF8; RILP* | TSS1500 | 3.55% | 4.40 | 1.08E-05 | 5.20E-02 |  |
| 23 | cg09801924 | 11 | 65425948 | *RELA* | Body | 1.23% | 4.51 | 6.51E-06 | 3.98E-02 |  |
| 24 | cg24135793 | 19 | 13122567 | *NFIX* | Body | 2.00% | -4.76 | 1.90E-06 | 2.51E-02 | [9, 11] |
| 25 | cg14718379 | 7 | 71806067 | *CALN1* | Body | 0.81% | -4.29 | 1.76E-05 | 6.31E-02 |  |
| 26 | cg01373932 | 6 | 31502462 | *BAT1* | Body | 2.62% | -3.98 | 6.84E-05 | 1.38E-01 |  |
| 27 | cg12680071 | 19 | 36139062 | *COX6B1* | TSS200 | 1.76% | -3.95 | 7.75E-05 | 1.42E-01 |  |
| 28 | cg01005506 | 10 | 64565768 | *ADO* | 1stExon | 1.64% | -4.07 | 4.65E-05 | 1.16E-01 |  |
| 29 | cg26103104 | 5 | 139490895 | *NA* | NA | 1.29% | -4.21 | 2.56E-05 | 8.11E-02 | [9] |
| 30 | cg22007110 | 7 | 30737599 | *NA* | NA | 0.57% | 3.97 | 7.20E-05 | 1.41E-01 | [10] |
| 31 | cg09284949 | 19 | 51190179 | *SHANK1* | Body | 0.84% | 3.92 | 8.69E-05 | 1.51E-01 |  |
| 32 | cg09635954 | 7 | 29605624 | *PRR15* | 5UTR | 0.80% | -4.42 | 9.82E-06 | 4.95E-02 |  |
| 33 | cg22499893 | 1 | 24307535 | *SFRS13A* | TSS1500 | 1.33% | -3.91 | 9.23E-05 | 1.53E-01 |  |
| 34 | cg13442969^LA^ | 12 | 68044208 | *DYRK2* | 5UTR | 4.87% | -6.68 | 2.42E-11 | 4.94E-06 | [9] |
| 35 | cg23728471 | 17 | 42345689 | *SLC4A1* | TSS200 | 1.47% | 4.00 | 6.25E-05 | 1.31E-01 |  |
| 36 | cg20732160 | 3 | 48590040 | *PFKFB4* | Body | 2.58% | -4.46 | 8.13E-06 | 4.29E-02 |  |
| 37 | cg01278873 | 19 | 44764101 | *ZNF233* | 5UTR | 1.40% | -4.39 | 1.14E-05 | 5.32E-02 |  |
| 38 | cg21550372 | 14 | 100908908 | *WDR25* | Body | 2.39% | -4.55 | 5.28E-06 | 3.82E-02 |  |
| 39 | cg00256932 | 22 | 51041732 | *MAPK8IP2* | 1stExon | 2.16% | 4.03 | 5.68E-05 | 1.26E-01 |  |
| 40 | cg03546163 | 6 | 35654363 | *FKBP5* | 5UTR | 1.01% | -4.39 | 1.14E-05 | 5.32E-02 |  |
| 41 | cg14395885 | 9 | 130700923 | *DPM2* | TSS200 | 0.73% | -4.62 | 3.87E-06 | 3.61E-02 |  |
| 42 | cg16423756 | 11 | 122526190 | *UBASH3B* | TSS1500 | 3.16% | 4.47 | 7.89E-06 | 4.29E-02 |  |
| 43 | cg00359465 | 1 | 2004346 | *PRKCZ* | TSS1500 | 1.77% | 4.01 | 6.01E-05 | 1.29E-01 |  |
| 44 | cg18487598 | 5 | 178735372 | *ADAMTS2* | Body | 1.93% | -4.01 | 6.11E-05 | 1.29E-01 |  |
| 45 | cg10381071 | 15 | 70391035 | *TLE3* | TSS1500 | 0.63% | -4.26 | 2.02E-05 | 6.98E-02 |  |
| 46 | cg12825509^LA^ | 3 | 185648568 | *TRA2B* | Body | 3.52% | -5.40 | 6.62E-08 | 3.00E-03 | [9, 11] |
| 47 | cg20970380 | 1 | 116676103 | *C1orf161* | 3UTR | 4.05% | -4.61 | 4.06E-06 | 3.68E-02 |  |
| 48 | cg02538135 | 19 | 2200125 | *DOT1L* | Body | 0.68% | 3.97 | 7.28E-05 | 1.41E-01 |  |
| 49 | cg07866212 | 8 | 23836952 | *NA* | NA | 1.82% | 4.35 | 1.37E-05 | 5.54E-02 |  |
| 50 | cg11704631 | 21 | 36395663 | *RUNX1* | Body | 2.96% | -4.84 | 1.29E-06 | 1.89E-02 | [9] |
| 51 | cg07769421 | 17 | 80816851 | *TBCD* | Body | 1.29% | 3.97 | 7.32E-05 | 1.41E-01 |  |
| 52 | cg24136700 | 17 | 17696044 | *RAI1* | 5UTR | 0.50% | 4.32 | 1.57E-05 | 6.00E-02 |  |
| 53 | cg23747342 | 12 | 25539794 | *NA* | NA | 0.99% | 4.48 | 7.37E-06 | 4.19E-02 |  |
| 54 | cg15690475 | 17 | 44101453 | *MAPT* | Body | 1.86% | 4.44 | 8.82E-06 | 4.55E-02 |  |
| 55 | cg26841068 | 1 | 203456691 | *PRELP* | 3UTR | 2.88% | -4.38 | 1.21E-05 | 5.37E-02 | [10, 11] |
| 56 | cg27653384 | 22 | 22293118 | *PPM1F* | Body | 0.47% | 4.36 | 1.33E-05 | 5.54E-02 | [9] |
| 57 | cg17953300 | 11 | 65418265 | *SIPA1* | 3UTR | 1.52% | 4.53 | 6.01E-06 | 3.90E-02 |  |
| 58 | cg25673668 | 8 | 895601 | *NA* | NA | 0.96% | -3.93 | 8.61E-05 | 1.51E-01 |  |
| 59 | cg03401875 | 5 | 178684658 | *ADAMTS2* | Body | 0.49% | -4.01 | 6.03E-05 | 1.29E-01 |  |
| 60 | cg21786227 | 1 | 154951605 | *CKS1B* | Body | 2.57% | -4.06 | 4.98E-05 | 1.18E-01 |  |
| 61 | cg19731612 | 5 | 176559334 | *NSD1* | TSS1500 | 1.84% | -5.08 | 3.69E-07 | 8.18E-03 | [9, 10] |
| 62 | cg10891521 | 17 | 81047941 | *METRNL* | Body | 1.04% | 4.68 | 2.93E-06 | 3.12E-02 |  |
| 63 | cg15636519 | 2 | 191894418 | *STAT4* | 3UTR | 1.55% | -4.13 | 3.63E-05 | 1.00E-01 |  |
| 64 | cg06937549 | 5 | 179046350 | *HNRNPH1* | Body | 2.33% | -4.56 | 5.05E-06 | 3.82E-02 |  |
| 65 | cg03404339 | 12 | 52639319 | *KRT7* | Body | 1.06% | -3.89 | 9.91E-05 | 1.56E-01 |  |
| 66 | cg10803871 | 5 | 174082675 | *NA* | NA | 1.45% | -3.96 | 7.60E-05 | 1.42E-01 |  |
| 67 | cg03163545 | 16 | 1593415 | *IFT140; TMEM204* | Body | 1.58% | 4.24 | 2.21E-05 | 7.49E-02 |  |
| 68 | cg18289746 | 6 | 112408679 | *TUBE1; C6orf225* | 5UTR | 1.64% | 3.91 | 9.08E-05 | 1.53E-01 |  |
| 69 | cg21194066 | 11 | 67052509 | *ADRBK1* | Body | 2.16% | -4.30 | 1.68E-05 | 6.15E-02 |  |
| 70 | cg01182455 | 9 | 131313189 | *NA* | NA | 2.62% | 3.96 | 7.35E-05 | 1.41E-01 |  |
| 71 | cg06232845 | 7 | 133625048 | *EXOC4* | Body | 1.03% | -4.07 | 4.62E-05 | 1.16E-01 |  |
| 72 | cg14259466 | 10 | 135090997 | *ADAM8* | TSS1500 | 1.46% | 4.51 | 6.56E-06 | 3.98E-02 |  |
| 73 | cg11697038 | 1 | 27931284 | *AHDC1* | TSS1500 | 1.51% | -4.10 | 4.13E-05 | 1.10E-01 |  |
| 74 | cg00294109 | 3 | 3219781 | *CRBN* | Body | 1.89% | 4.96 | 6.91E-07 | 1.18E-02 |  |
| 75 | cg09482421 | 6 | 27470541 | *NA* | NA | 0.66% | 3.95 | 7.73E-05 | 1.42E-01 |  |
| 76 | cg01881182 | 16 | 8806531 | *ABAT* | TSS1500 | 2.70% | -4.07 | 4.67E-05 | 1.16E-01 |  |
| 77 | cg10035272 | 6 | 142467043 | *VTA1* | TSS1500 | 1.86% | -4.15 | 3.34E-05 | 9.48E-02 |  |
| 78 | cg19939130 | 1 | 158978468 | *IFI16* | TSS1500 | 2.21% | -4.51 | 6.52E-06 | 3.98E-02 |  |
| 79 | cg02633398 | 16 | 88974944 | *CBFA2T3* | 5UTR | 1.17% | 3.96 | 7.66E-05 | 1.42E-01 |  |
| 80 | cg20699548^LA^ | 8 | 71060638 | *NCOA2* | Body | 2.71% | -4.49 | 7.12E-06 | 4.15E-02 | [9] |
| 81 | cg27017993 | 14 | 74814040 | *C14orf115* | TSS1500 | 3.00% | 3.96 | 7.42E-05 | 1.41E-01 |  |
| 82 | cg08250921 | 16 | 88111009 | *NA* | NA | 3.20% | 4.69 | 2.76E-06 | 3.12E-02 |  |
| 83 | cg26284735 | 3 | 5065165 | *NA* | NA | 2.21% | 3.97 | 7.17E-05 | 1.41E-01 |  |
| 84 | cg15705813 | 2 | 70297499 | *NA* | NA | 1.78% | -5.30 | 1.14E-07 | 3.87E-03 | [9, 10] |
| 85 | cg15033653 | 12 | 113587581 | *CCDC42B* | TSS200 | 3.14% | 4.62 | 3.89E-06 | 3.61E-02 |  |
| 86 | cg26340050 | 14 | 105771879 | *NA* | NA | 3.41% | 4.56 | 5.08E-06 | 3.82E-02 |  |
| 87 | cg27155460 | 10 | 45420821 | *TMEM72* | Body | 2.70% | 4.44 | 8.91E-06 | 4.55E-02 |  |
| 88 | cg13866253 | 11 | 77093001 | *PAK1* | Body | 2.02% | -5.31 | 1.12E-07 | 3.87E-03 |  |
| 89 | cg23352030 | 20 | 62198469 | *PRIC285* | 1stExon | 1.14% | 4.38 | 1.18E-05 | 5.35E-02 |  |
| 90 | cg19536127 | 2 | 47404286 | *CALM2* | TSS1500 | 1.79% | 4.35 | 1.38E-05 | 5.54E-02 |  |
| 91 | cg01156249 | 16 | 4714794 | *MGRN1* | Body | 3.76% | 4.27 | 1.99E-05 | 6.98E-02 |  |
| 92 | cg04978583 | 1 | 12091409 | *MIIP* | Body | 2.69% | 4.01 | 5.95E-05 | 1.29E-01 |  |
| 93 | cg18121224 | 5 | 176559563 | *NSD1* | TSS1500 | 2.05% | -4.21 | 2.59E-05 | 8.15E-02 | [10] |
| 94 | cg00407659 | 5 | 150538414 | *ANXA6* | TSS1500 | 1.04% | -3.91 | 9.18E-05 | 1.53E-01 | [10] |
| 95 | cg08695062 | 5 | 53813260 | *SNX18* | TSS1500 | 1.42% | 3.94 | 8.06E-05 | 1.47E-01 |  |
| 96 | cg02314339 | 10 | 91020653 | *NA* | NA | 2.78% | -4.18 | 2.87E-05 | 8.67E-02 | [9, 10] |
| 97 | cg03887584 | 10 | 98130310 | *TLL2* | Body | 2.11% | 4.05 | 5.13E-05 | 1.20E-01 |  |
| 98 | cg08307171 | 19 | 1957775 | *CSNK1G2* | 5UTR | 1.23% | 4.30 | 1.69E-05 | 6.15E-02 |  |
| 99 | cg21845080 | 3 | 196065306 | *TM4SF19* | TSS200 | 0.44% | 4.47 | 7.76E-06 | 4.29E-02 |  |
| 100 | cg07912416 | 6 | 30860130 | *DDR1* | Body | 1.34% | 4.01 | 6.03E-05 | 1.29E-01 |  |
| 101 | cg13547518 | 2 | 2653590 | *NA* | NA | 0.92% | 4.06 | 4.82E-05 | 1.17E-01 |  |
| 102 | cg10603800 | 17 | 80408909 | *C17orf62* | TSS1500 | 0.96% | 4.08 | 4.59E-05 | 1.16E-01 |  |
| 103 | cg11494699 | 11 | 36588217 | *RAG1* | TSS1500 | 1.25% | 3.90 | 9.68E-05 | 1.56E-01 |  |
| 104 | cg13966547 | 1 | 2406284 | *PLCH2* | TSS1500 | 1.21% | -4.57 | 4.79E-06 | 3.82E-02 |  |
| 105 | cg25746394 | 19 | 45450501 | *APOC2* | 5UTR | 1.61% | 4.08 | 4.56E-05 | 1.16E-01 |  |
| 106 | cg15326297 | 2 | 235464680 | *NA* | NA | 2.25% | 3.91 | 9.38E-05 | 1.53E-01 |  |
| 107 | cg22537604 | 19 | 43857074 | *CD177* | TSS1500 | 2.08% | -4.49 | 7.31E-06 | 4.19E-02 |  |
| 108 | cg10361922 | 17 | 40925790 | *VPS25* | Body | 1.92% | -4.19 | 2.75E-05 | 8.45E-02 |  |
| 109 | cg13341668 | 3 | 50359909 | *HYAL2* | TSS1500 | 1.55% | -3.91 | 9.34E-05 | 1.53E-01 |  |
| 110 | cg11899596 | 8 | 145663791 | *NFKBIL2* | Body | 0.46% | 3.98 | 6.85E-05 | 1.38E-01 |  |
| 111 | cg03394159 | 8 | 29197844 | *DUSP4* | Body | 1.90% | 4.42 | 1.01E-05 | 4.97E-02 |  |
| 112 | cg20414364 | 5 | 1608614 | *LOC728613* | Body | 0.87% | 5.47 | 4.56E-08 | 2.66E-03 |  |
| 113 | cg19525078 | 12 | 29581042 | *OVCH1* | Body | 2.72% | 4.09 | 4.21E-05 | 1.12E-01 | [10] |
| 114 | cg22496559 | 3 | 196065318 | *TM4SF19* | TSS200 | 0.77% | 4.12 | 3.80E-05 | 1.03E-01 |  |
| 115 | cg01628467 | 17 | 7037406 | *NA* | NA | 1.32% | -4.16 | 3.21E-05 | 9.31E-02 |  |
| 116 | cg19821589 | 2 | 170591199 | *KLHL23* | 5UTR | 1.34% | -4.07 | 4.80E-05 | 1.17E-01 |  |
| 117 | cg06059663 | 1 | 245319431 | *KIF26B* | Body | 2.12% | -4.59 | 4.45E-06 | 3.77E-02 |  |
| 118 | cg14014731 | 9 | 19378679 | *RPS6* | Body | 1.06% | -3.98 | 6.90E-05 | 1.38E-01 |  |
| 119 | cg01017397 | 7 | 955497 | *ADAP1* | Body | 1.38% | 4.35 | 1.36E-05 | 5.54E-02 |  |
| 120 | cg26829071^LA^ | 12 | 131590596 | *GPR133* | Body | 1.72% | 3.94 | 8.21E-05 | 1.48E-01 | [9] |
| 121 | cg01877260 | 15 | 38845483 | *RASGRP1* | Body | 1.87% | -3.94 | 8.18E-05 | 1.48E-01 |  |
| 122 | cg08204159 | 19 | 54642290 | *CNOT3* | 5UTR | 1.27% | -3.89 | 9.87E-05 | 1.56E-01 |  |
| 123 | cg06825661 | 18 | 13260398 | *C18orf1* | 5UTR | 1.56% | 3.91 | 9.14E-05 | 1.53E-01 |  |
| 124 | cg11302401 | 6 | 6688847 | *NA* | NA | 2.63% | -4.59 | 4.52E-06 | 3.77E-02 |  |
| 125 | cg26687579 | 1 | 2243111 | *NA* | NA | 2.42% | 3.96 | 7.36E-05 | 1.41E-01 |  |
| 126 | cg22503354 | 12 | 7341644 | *PEX5* | TSS1500 | 2.30% | 4.12 | 3.70E-05 | 1.02E-01 |  |
| 127 | cg18595324 | 1 | 31838695 | *FABP3* | 3UTR | 2.56% | -4.09 | 4.25E-05 | 1.12E-01 |  |
| 128 | cg22954438 | 3 | 23548050 | *UBE2E2* | Body | 1.59% | 3.93 | 8.65E-05 | 1.51E-01 |  |
| 129 | cg20283107 | 8 | 124788969 | *FAM91A1* | Body | 2.73% | -4.28 | 1.87E-05 | 6.65E-02 |  |
| 130 | cg13836098 | 6 | 26225268 | *HIST1H3E* | TSS200 | 1.68% | -4.07 | 4.72E-05 | 1.17E-01 |  |
| 131 | cg23684449 | 16 | 46919194 | *GPT2* | 5UTR | 1.52% | -4.38 | 1.18E-05 | 5.35E-02 | [9, 11] |
| 132 | cg12474798 | 10 | 64565772 | *ADO* | 1stExon | 1.17% | -3.90 | 9.71E-05 | 1.56E-01 | [10] |
| 133 | cg18068637 | 5 | 168245286 | *SLIT3* | Body | 1.20% | -4.16 | 3.12E-05 | 9.28E-02 |  |
| 134 | cg11791144 | 8 | 32129319 | *NRG1* | Body | 1.61% | -3.93 | 8.62E-05 | 1.51E-01 |  |
| 135 | cg02346737 | 5 | 142490096 | *ARHGAP26* | Body | 1.23% | -3.91 | 9.12E-05 | 1.53E-01 |  |
| 136 | cg15329179 | 5 | 131993728 | *IL13* | TSS200 | 2.12% | 4.31 | 1.64E-05 | 6.11E-02 |  |
| 137 | cg06549901 | 16 | 55851316 | *CES1* | Body | 2.95% | -3.98 | 6.89E-05 | 1.38E-01 |  |
| 138 | cg01022780 | 3 | 48598236 | *NA* | NA | 1.10% | 3.90 | 9.50E-05 | 1.55E-01 |  |
| 139 | cg18034719 | 5 | 176860863 | *GRK6* | Body | 2.55% | 4.22 | 2.46E-05 | 7.97E-02 |  |
| 140 | cg11724472 | 2 | 12249222 | *NA* | NA | 1.86% | 4.00 | 6.22E-05 | 1.31E-01 |  |
| 141 | cg11599718 | 12 | 123357128 | *VPS37B* | Body | 1.29% | 4.53 | 5.89E-06 | 3.90E-02 |  |
| 142 | cg10692140 | 6 | 30496072 | *NA* | NA | 1.34% | -4.53 | 5.83E-06 | 3.90E-02 |  |
| 143 | cg02171825 | 1 | 26517586 | *CATSPER4* | Body | 1.21% | 3.92 | 8.71E-05 | 1.51E-01 |  |
| ^LA^The CpGs that are selected using LASSO in Liu C, Marioni RE, Hedman ÅK, Pfeiffer L, Tsai PC, Reynolds LM et al. A DNA methylation biomarker of alcohol consumption. *Molecular psychiatry* 2018 Feb; **23(2)**:422. | | | | | | | | | | |

**Supplementary Fig. S1.** Correlation between the natural logarithm of Phosphatidylethanol (ln(PEth)) and Alcohol Use Disorders Identification Test-Consumption (AUDIT-C, first 3 items of AUDIT) score in Cohort 1. **a.** Scatter plot showing significant association between the ln(PEth) value and the AUDIT-C score (The Pearson correlation between ln(PEth) and AUDIT-C is 0.45 (95% CI: 0.39, 0.51) with $p$ < 2.00E-16); **b.** Violin plot showing significant difference of the ln(PEth) value between non-Hazardous Alcohol Drinking (non-HAD) (AUDIT-C < 4) participants and HAD (AUDIT-C >= 4) participants. The P-value of Welch’s t-test (degrees of freedom = 470) for non-HAD and HAD is 3.47E-33, which indicates that the biomarker PEth and alcohol consumption are significantly correlated.


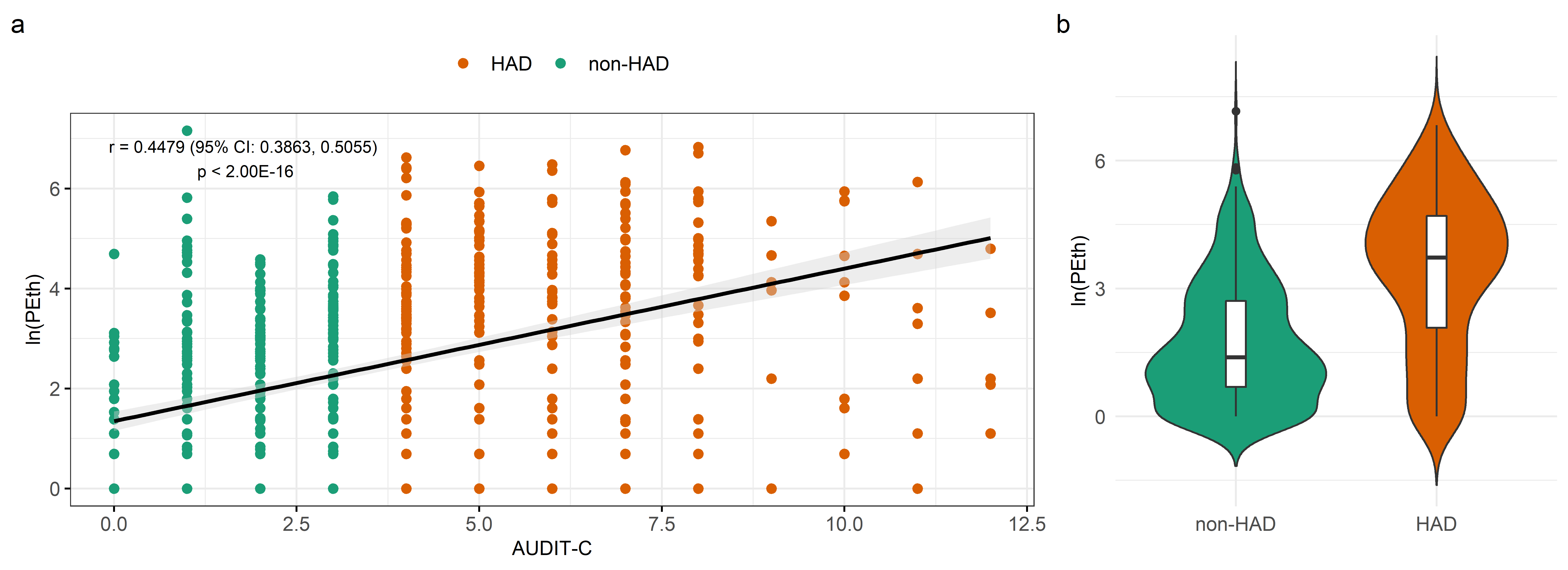


**Supplementary Fig. S2.** Correlation plots show the correlation between the top 30 principal components on DNA methylation (X-axis) and confounding factors including six cell types (Y-axis). **a**. without adjustment of confounding factors; **b**. with adjustment of confounding factors.





**Supplementary Fig. S3.** Manhattan plot and quantile-quantile (QQ) plot for the discovery set of Cohort 1. **a.** Manhattan plot of the chromosomal locations of $-\log_{10} (p)$ for the epigenome-wide association in 437,722 CpGs among the 580 males in the discovery sample set. The red line represents the threshold for Bonferroni-corrected p-value. The blue line represents the threshold for Benjamini–Hochberg false discovery rate (FDR)-corrected p-value; **b.** QQ plot for association at all 437,722 CpGs. $\lambda=1.086$ in the discovery epigenome-wide association analysis.


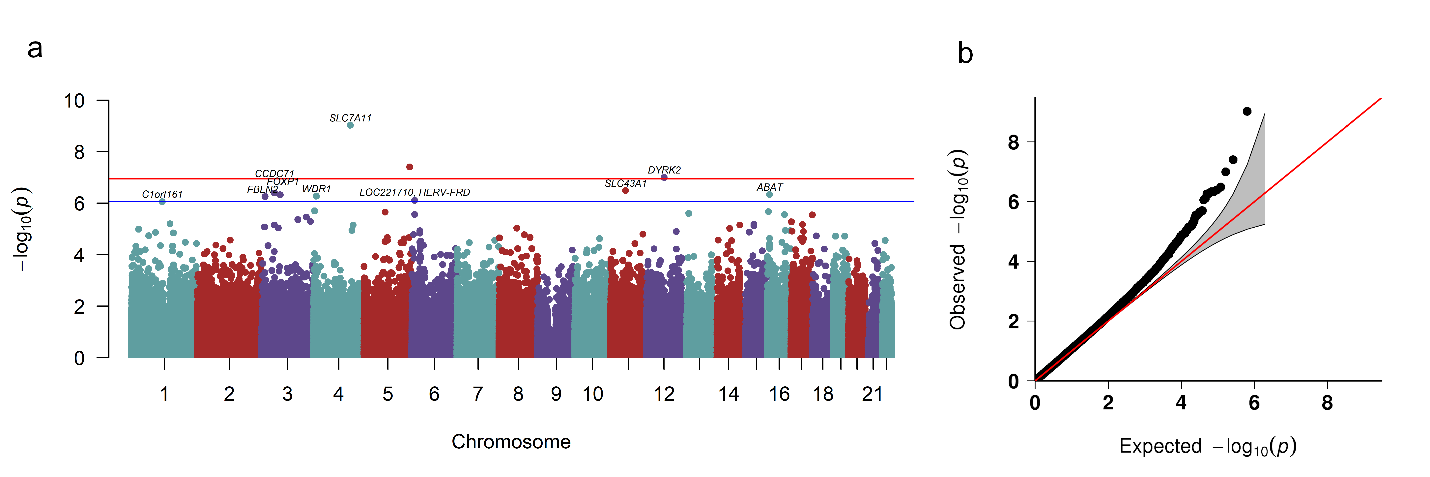


**Supplementary Fig. S4.** Manhattan plot and quantile-quantile (QQ) plot for the replication set of Cohort 1. **a.** Manhattan plot of the chromosomal locations of $-\log_{10} (p)$ for the epigenome-wide association in 846,604 CpGs among the 467 males in the replication sample set. The red line represents the threshold for Bonferroni-corrected p-value. The blue line represents the threshold for Benjamini–Hochberg false discovery rate (FDR)-corrected p-value; **b.** QQ plot for the association at all 846,604 CpGs. $\lambda=1.102$ in the replication epigenome-wide association analysis.


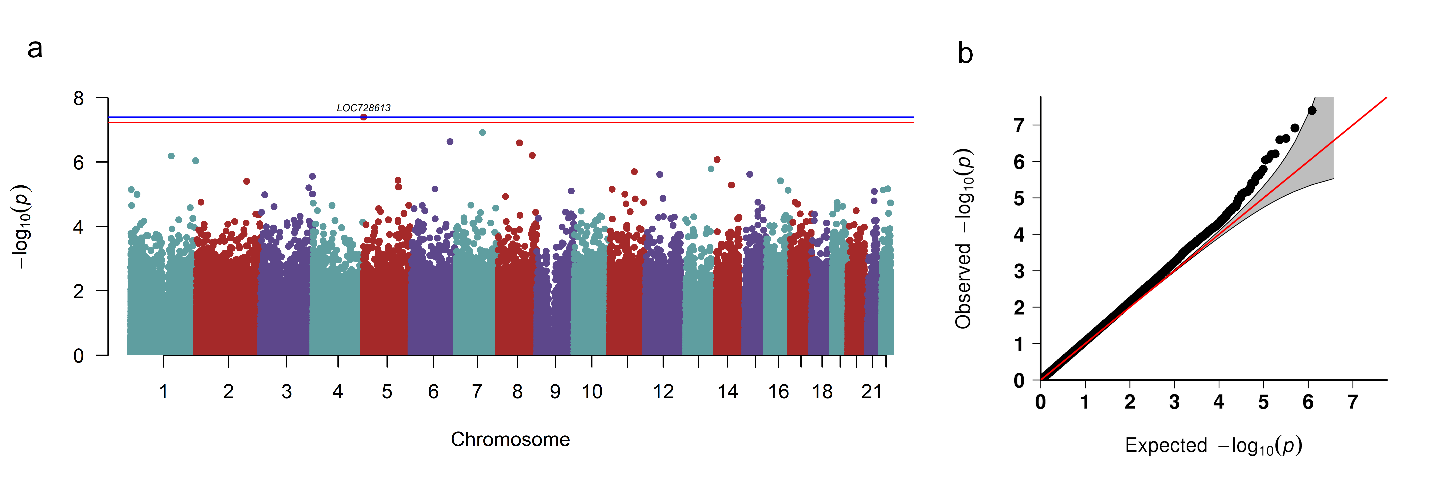


**Supplementary Fig. S5.** Quantile-Quantile (QQ) plot for meta-analysis and combined analysis. **a**. QQ plot for meta-analysis of epigenome-wide association studies (EWAS) (λ=1.130); **b**. QQ plot for combined analysis (EWAS conducted by combining the discovery and replication samples) (λ=1.442).


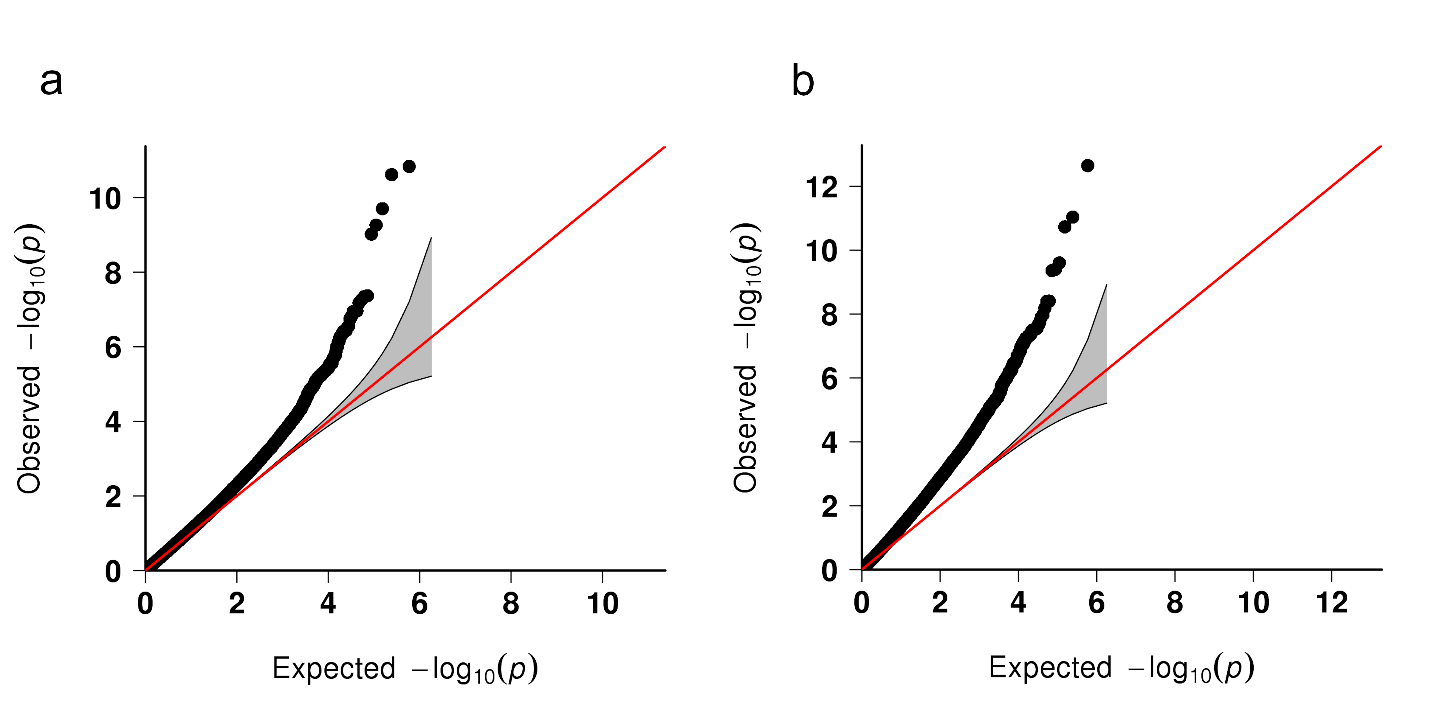


**Supplementary Fig. S6.** Receiver Operating Characteristic (ROC) curves of Phosphatidylethanol (PEth) with and without DNA methylation for predicting Hazardous Alcohol Drinking (HAD). ROC curve for predicting HAD by PEth alone, PEth with 12 CpGs (Bonferroni corrected p-value less than 5.00E-02), and PEth with 83 CpGs (Benjamini–Hochberg false discovery rate (FDR)-corrected p-value less than 5.00E-02) for samples in Cohort 1.


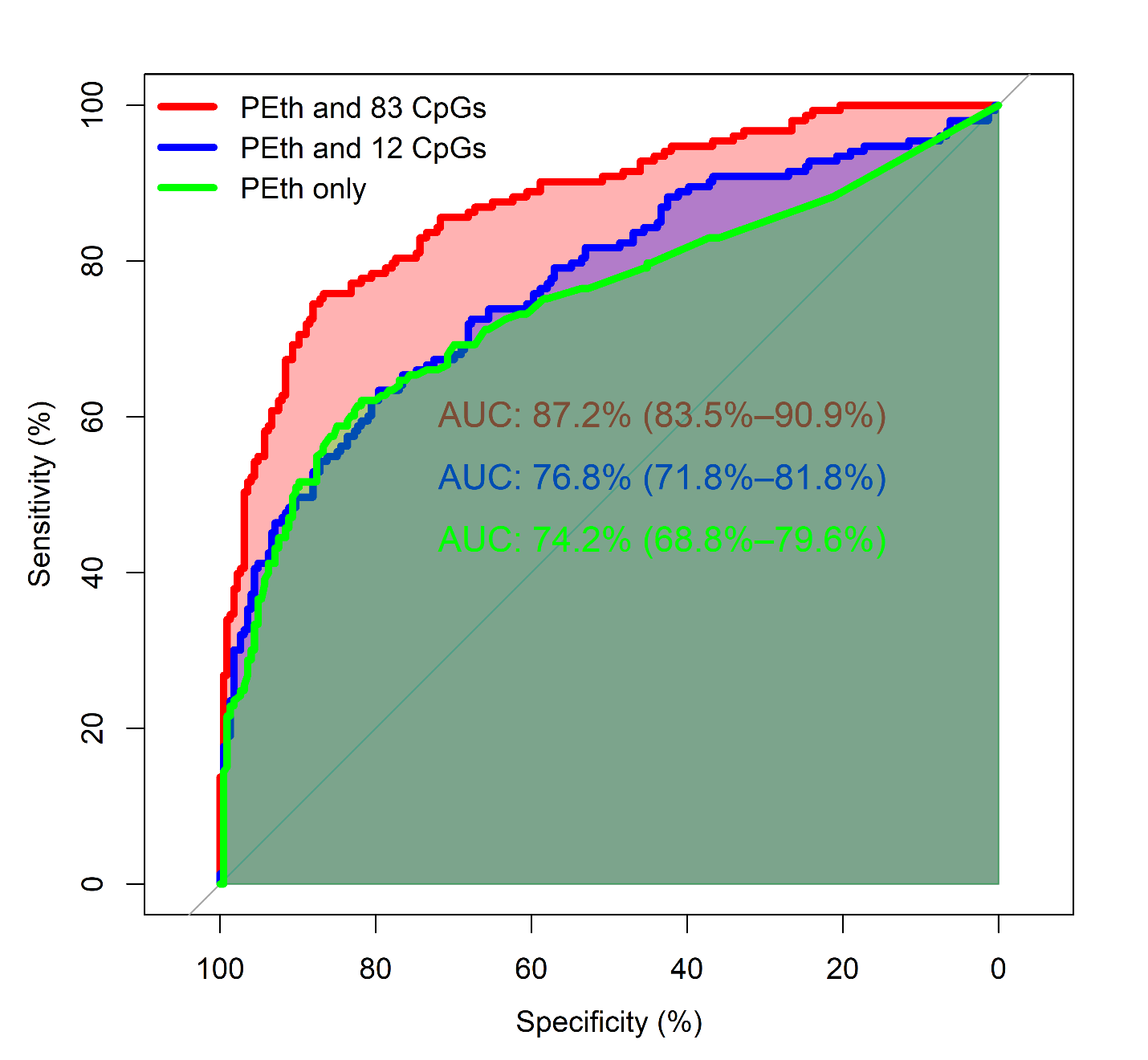


**Supplementary Fig. S7.** Correlation between PolyGenic Methylation Score (PGMS) and alcohol-related phenotypes. Here, PGMS was constructed by 83 Phosphatidylethanol (PEth)-related CpGs. **a.** Scatterplots of Alcohol Use Disorders Identification Test (AUDIT) score and PGMS; **b.** Scatterplots of Alcohol Use Disorders Identification Test-Consumption items (AUDIT-C) and PGMS; **c.** Scatterplots of Alcohol Use Disorders Identification Test-Problem items (AUDIT-P) score and PGMS.


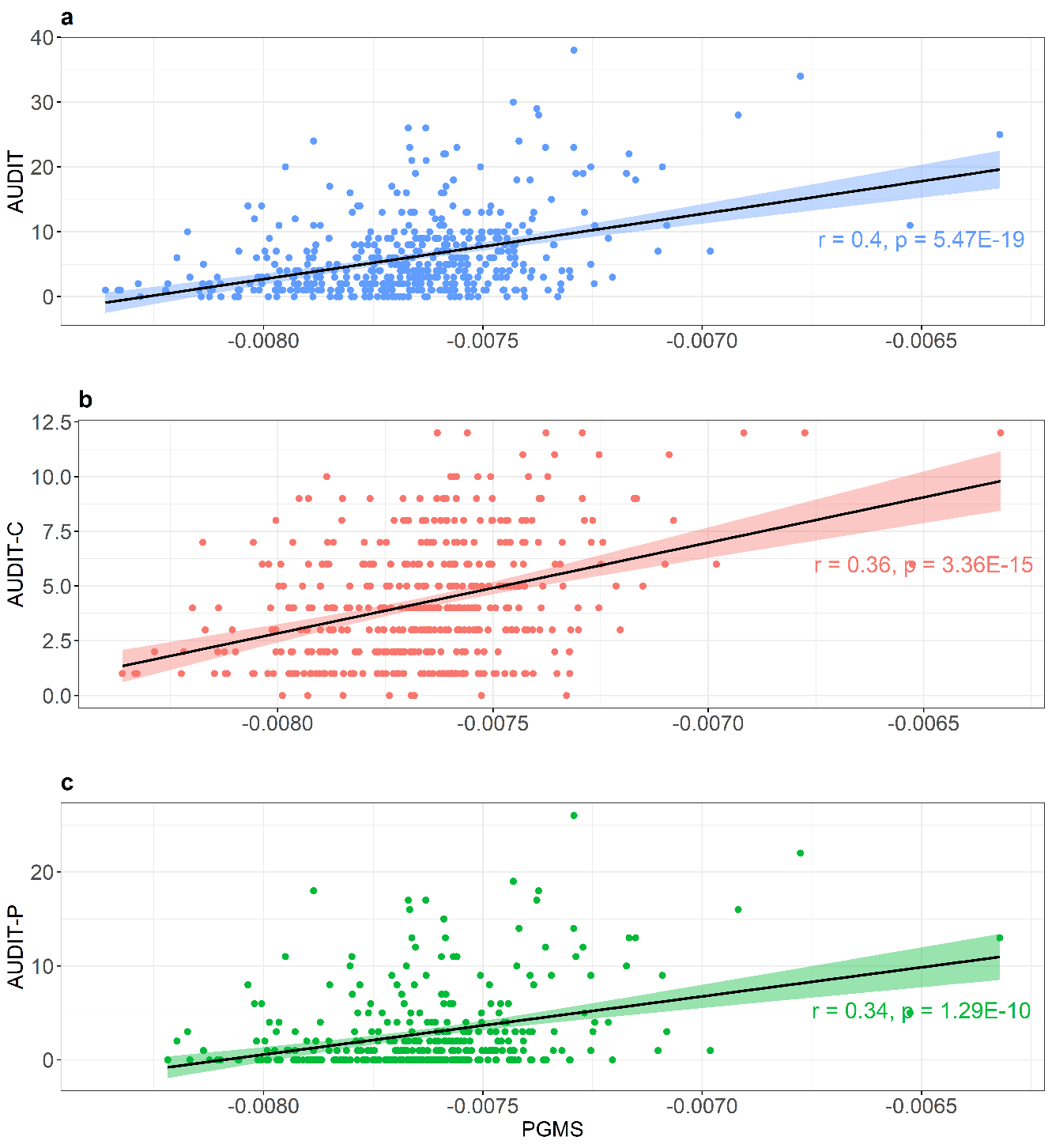


**Supplementary Fig. S8.** Sensitivity test of the preselected CpG set with different cutoff values (1E-06, 1E-05, 1E-04, and 1E-03). Red line: the area under the receiver operating characteristic curve (AUC); Blue line: the difference in adjusted $R^{2}$ between model with predicted variable and model without predicted variable (incremental $R^{2}$) using all samples in Cohort 2.


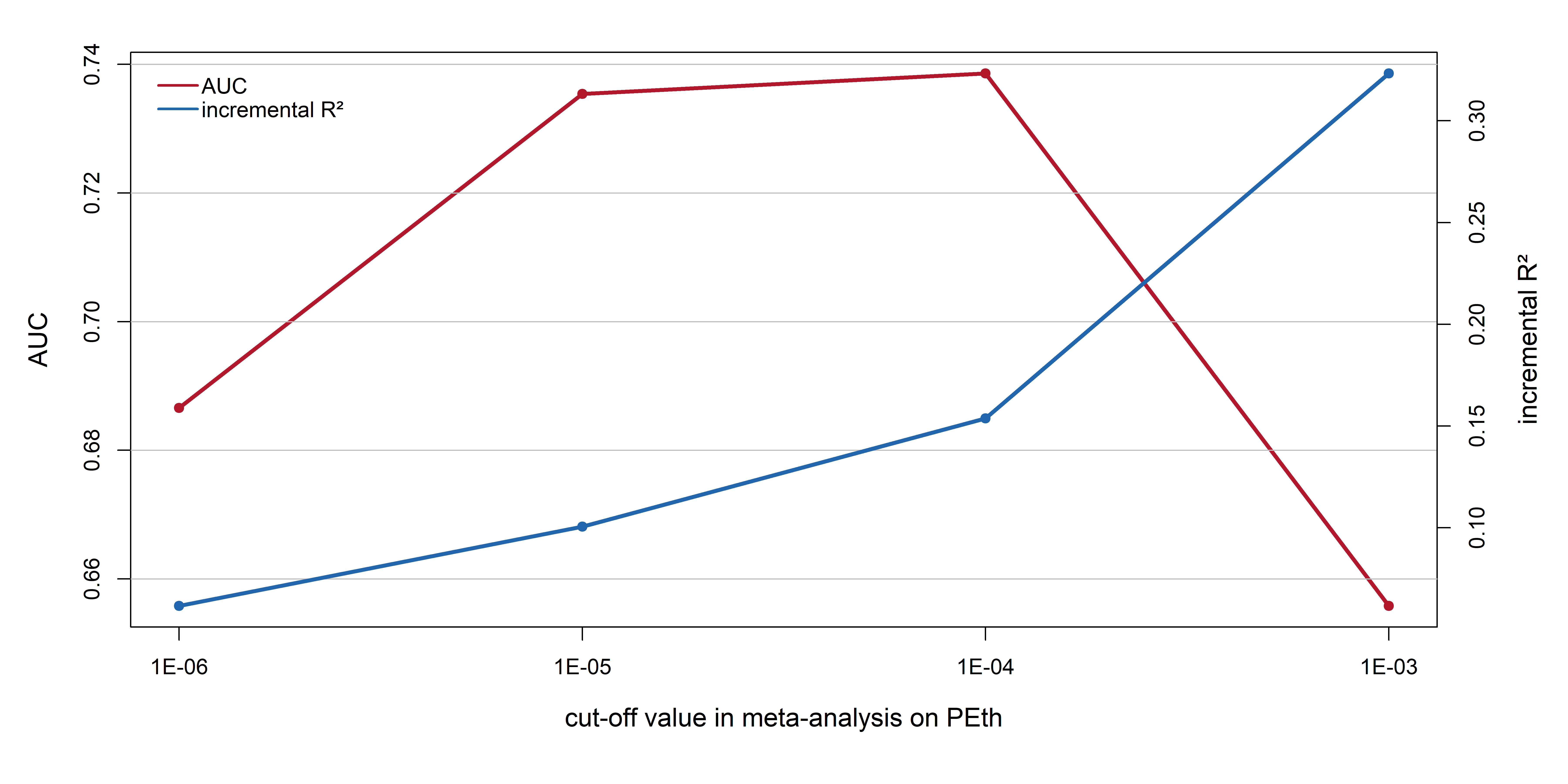


**Supplementary Fig. S9.** Database for Annotation, Visualization and Integrated Discovery (DAVID) pathway analysis for the 143 CpGs selected by elastic net regularization (ENR) with $p<$1E-03.


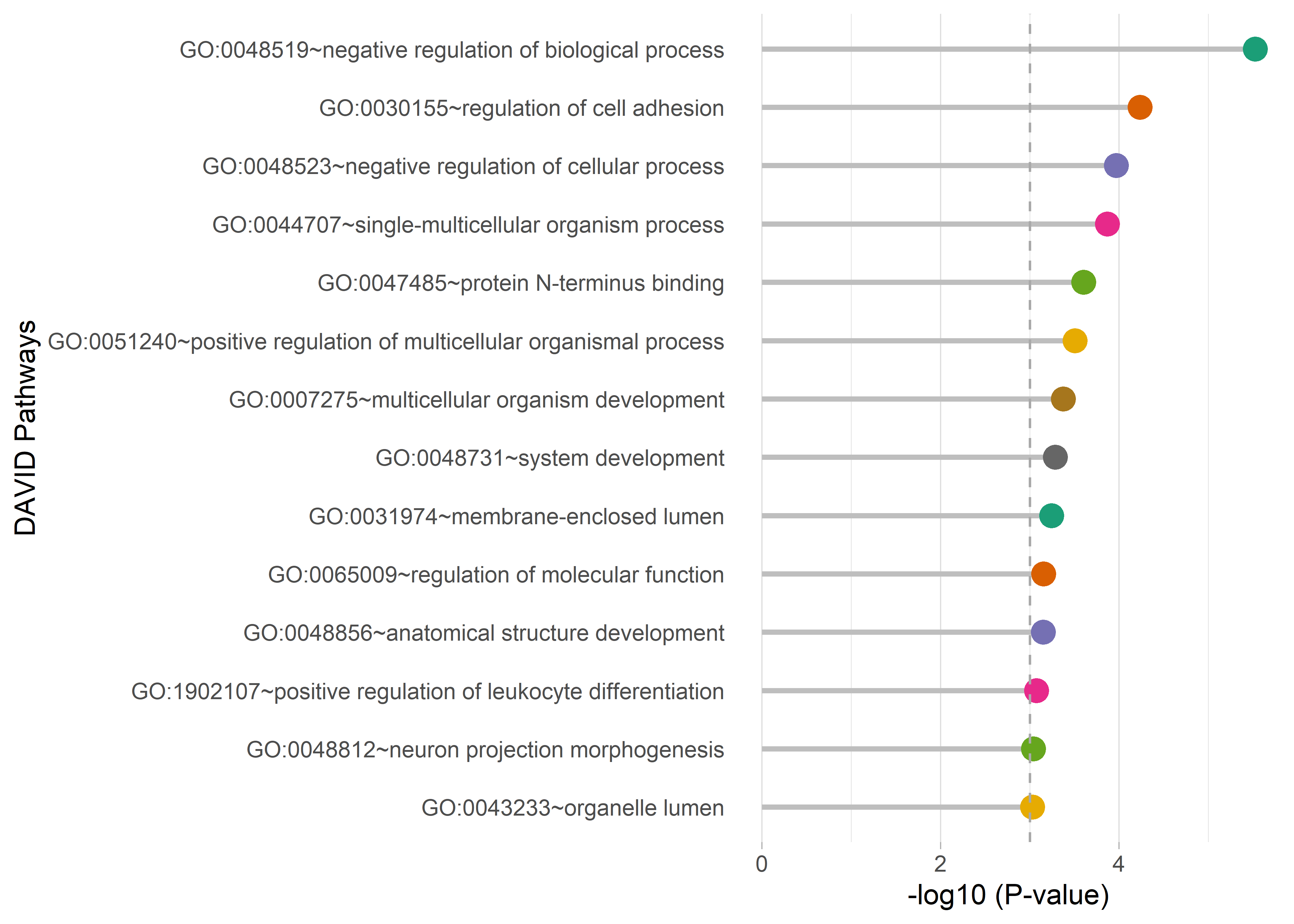


**Supplementary Fig. S10.** Comparison of the identified CpG sets for alcohol consumption between the present study and Liu et al (2018) A DNA methylation biomarker of alcohol consumption. *Molecular Psychiatry*, **23 (2)**; 422. ENR: elastic net regularization; EWS: epigenome-wide significant; AA: African Americans; ML: Machine Learning.


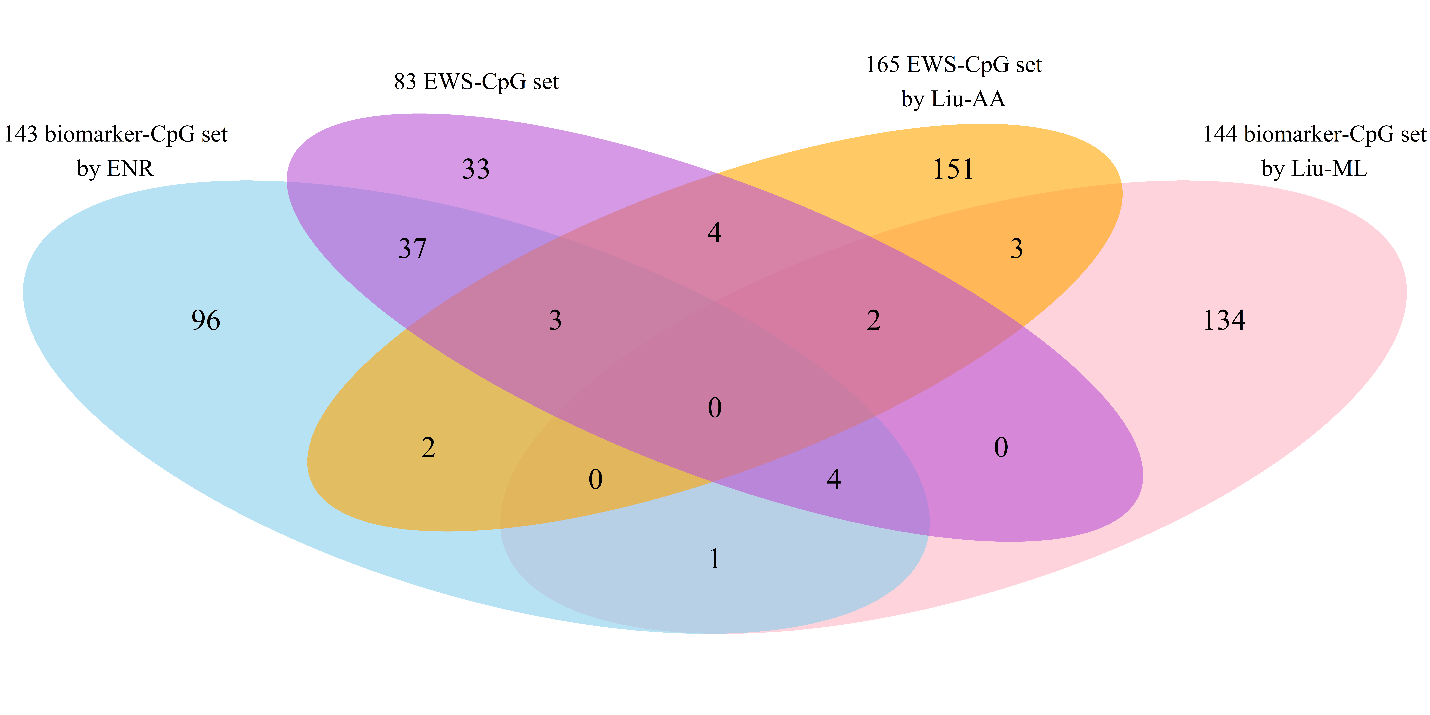


# **References**

1. Zhang X, Hu Y, Justice AC, Li B, Wang Z, Zhao H *et al.* DNA methylation signatures of illicit drug injection and hepatitis C are associated with HIV frailty. *Nat Commun* 2017; **8**(1)**:** 2243.

2. Lehne B, Drong AW, Loh M, Zhang W, Scott WR, Tan S-T *et al.* A coherent approach for analysis of the Illumina HumanMethylation450 BeadChip improves data quality and performance in epigenome-wide association studies. *Genome biology* 2015; **16**(1)**:** 37.

3. Houseman EA, Accomando WP, Koestler DC, Christensen BC, Marsit CJ, Nelson HH *et al.* DNA methylation arrays as surrogate measures of cell mixture distribution. *BMC Bioinformatics* 2012; **13:** 86.

4. Jaffe AE, Irizarry RA. Accounting for cellular heterogeneity is critical in epigenome-wide association studies. *Genome Biol* 2014; **15**(2)**:** R31.

5. Jung M, Pfeifer GP. Aging and DNA methylation. *BMC biology* 2015; **13**(1)**:** 7.

6. Joubert BR, Håberg SE, Nilsen RM, Wang X, Vollset SE, Murphy SK *et al.* 450K epigenome-wide scan identifies differential DNA methylation in newborns related to maternal smoking during pregnancy. *Environmental health perspectives* 2012; **120**(10)**:** 1425-1431.

7. Zhang FF, Cardarelli R, Carroll J, Fulda KG, Kaur M, Gonzalez K *et al.* Significant differences in global genomic DNA methylation by gender and race/ethnicity in peripheral blood. *Epigenetics* 2011; **6**(5)**:** 623-629.

8. Jiao C, Zhang C, Dai R, Xia Y, Wang K, Giase G *et al.* Positional effects revealed in Illumina methylation array and the impact on analysis. *Epigenomics* 2018; **10**(5)**:** 643-659.

9. Liu C, Marioni RE, Hedman ÅK, Pfeiffer L, Tsai P-C, Reynolds LM *et al.* A DNA methylation biomarker of alcohol consumption. *Molecular psychiatry* 2018; **23**(2)**:** 422.

10. Wilson LE, Xu Z, Harlid S, White AJ, Troester MA, Sandler DP *et al.* Alcohol and DNA Methylation: An Epigenome-Wide Association Study in Blood and Normal Breast Tissue. *American journal of epidemiology* 2019; **188**(6)**:** 1055-1065.

11. Dugué P-A, Wilson R, Lehne B, Jayasekara H, Wang X, Chol-Hee J *et al.* Alcohol consumption is associated with widespread changes in blood DNA methylation: analysis of cross-sectional and longitudinal data. *bioRxiv* 2018**:** 452953.
